# Light exposure and sleep architecture in real-world settings

**DOI:** 10.1038/s44323-026-00087-z

**Published:** 2026-07-10

**Authors:** Sena Gulsum Akgun, Burcu Gemici, Chloe Roddis, Lucien Bickerstaff, Beatriz Bano Otalora, Nina Milosavljevic, Timothy M. Brown, Robert J. Lucas, Altug Didikoglu

**Affiliations:** 1 Department of Neuroscience, Izmir Institute of Technology, Gulbahce, Urla Izmir, Turkey; 2 Centre for Biological Timing, Division of Neuroscience, School of Biological Sciences, Faculty of Biology Medicine and Health, University of Manchester, Manchester, UK; 3 Max Planck Institute for Biological Cybernetics, Translational Sensory & Circadian Neuroscience, Tübingen, Germany; 4 TUM School of Medicine and Health, Technical University of Munich, Munich, Germany; 5 Centre for Biological Timing, Division of Diabetes Endocrinology and Gastroenterology, School of Medical Sciences, Faculty of Biology Medicine and Health, University of Manchester, Manchester, UK

**Keywords:** Neuroscience, Psychology, Psychology

## Abstract

Light exposure can influence sleep timing, quality, and architecture, yet evidence from real-world settings remains limited. We investigated personal light exposure, quantified as melanopic equivalent daylight illuminance, and sleep parameters using consumer-grade wearables in a convenience sample of UK adults (*n* = 89). Participants simultaneously wore light sensors and sleep trackers for 7 days, yielding over 500 days of data, complemented by daily sleep diaries. Earlier sleep/wake timing was associated with longer-duration daytime light exposure, as well as more regular, less fragmented light patterns across the week. Higher interdaily stability and lower intradaily variability of light exposure were further linked to enhanced deep sleep intensity during the initial portion of the night. Subjective and wearable-derived measures were generally correlated, although disrupted sleep architecture was associated with greater discrepancies between subjective and wearable sleep estimates. These findings demonstrate the feasibility of naturalistic protocols and highlight the potential importance of brighter days and more stable, less fragmented light exposure patterns for sleep.

## Introduction

Sleep is a fundamental biological process, and its disruption is associated with a range of adverse health outcomes, including metabolic, cardiovascular, psychiatric, neurological, and cognitive disorders^1–5^. The circadian pacemaker, located in the suprachiasmatic nuclei (SCN) of the hypothalamus, interacts with the sleep homeostatic system to regulate consolidated periods of wakefulness and sleep aligned with the day–night cycle^6^. Light, acting through melanopsin-expressing intrinsically photosensitive retinal ganglion cells (ipRGCs), plays a critical role in both circadian and sleep regulation^7,8^. It resets the circadian clock and triggers acute physiological responses such as melatonin suppression and increased alertness^9,10^. Additionally, light can influence sleep through circadian-independent pathways by regulating preoptic area circuits that promote non-REM sleep without affecting REM sleep^11^.

Recent studies confirm that light exposure profoundly influences sleep architecture and circadian timing. Exposure to bright light prior to sleep delays the circadian phase^12,13^. Meta-analyses of laboratory studies show that evening light exposure increases sleep onset latency, reduces sleep efficiency, and tends to decrease the amount of slow-wave sleep compared to dim light conditions^14^. Light exposure during sleep further impairs sleep continuity, leading to greater fragmentation and alterations in sleep stage distribution^15^. In contrast, bright light exposure during the daytime, particularly in the early part of the day, reinforces circadian entrainment and improves subsequent sleep quality. Individuals who receive earlier and more intense daytime light tend to exhibit earlier circadian phase, greater sleep efficiency, reduced sleep fragmentation, lower proportion of REM sleep, greater accumulation of slow-wave sleep, improved subjective sleep quality, and a reduced risk of sleep disorders^7,16–22^.

While laboratory-based research has provided valuable insights into the effects of light on sleep and circadian rhythms, it often fails to fully capture the complexity of real-world conditions, such as dynamic ambient lighting, individual sleep preferences, and sociocultural influences^23^. These uncontrolled, ecologically valid variables can significantly confound outcomes. Consequently, there is growing recognition of the value of ecological momentary assessment approaches that incorporate both subjective self-reports and non-invasive wearable sensors to measure sleep and light exposure in naturalistic settings^24,25^. Studies employing actigraphy in combination with illuminance sensors have shown that greater light exposure during or before sleep and lower exposure during daytime are associated with later sleep and wake times, longer sleep onset latency, increased sleep fragmentation, and reduced sleep efficiency^13,26–29^.

Melanopic equivalent daylight illuminance (melanopic EDI) is a standardized metric that quantifies the effectiveness of light in stimulating ipRGCs, which play a key role in regulating non-visual physiological processes^30^. Consensus-based recommendations for indoor light exposure to support circadian physiology propose near-darkness during sleep, limiting exposure to less than 10 lux melanopic EDI in the 3 h prior to bedtime, and achieving daytime exposure exceeding 250 lux melanopic EDI^31^. Our previous research in naturalistic settings has indicated that higher melanopic EDI during the daytime and lower levels at night are associated with earlier sleep timing and reduced sleep onset latency^32^. However, real-world data quantifying light exposure in melanopic EDI terms and examining its associations with wearable sleep outcomes, including sleep staging, remain limited. The present study was designed to address this gap by examining the associations between real-world melanopic light exposure patterns and sleep architecture measured using wearable devices. We specifically tested the hypothesis that melanopic light exposure would be associated with wearable-derived sleep characteristics, including sleep stage distribution. Additionally, we investigated potential discrepancies between subjective and wearable sleep estimates and hypothesized that light exposure and sleep characteristics may contribute to these differences under naturalistic conditions.

## Results

### Measuring personal light exposure in real-world settings

We aimed to collect wearable sleep and personal light exposure data, measured in melanopic EDI, in naturalistic everyday life settings to investigate their relationship during regular human activity. Sleep was monitored using a wearable wristband Fitbit Charge 5 (Fitbit Inc., San Francisco, CA), while light exposure was simultaneously recorded using one of two wrist-worn devices: Spectrawear^33^ (University of Manchester) and ActLumus (Condor Instruments, Brazil). A total of 89 participants wore these devices concurrently. Over the 542-day data collection period, there was comprehensive coverage of the local photoperiod throughout the year in the study location (Manchester, UK). On average, participants contributed 6.1 valid days (SD = 1.2; range: 2–12) of data within the 7-day protocol, reflecting strong adherence to the study procedures. In a supplementary analysis, both light-logging devices demonstrated strong correlations with each other and with calibrated light sources, suggesting that potential confounding effects due to device differences are likely minimal (Fig. S1).

**Fig. 1:**
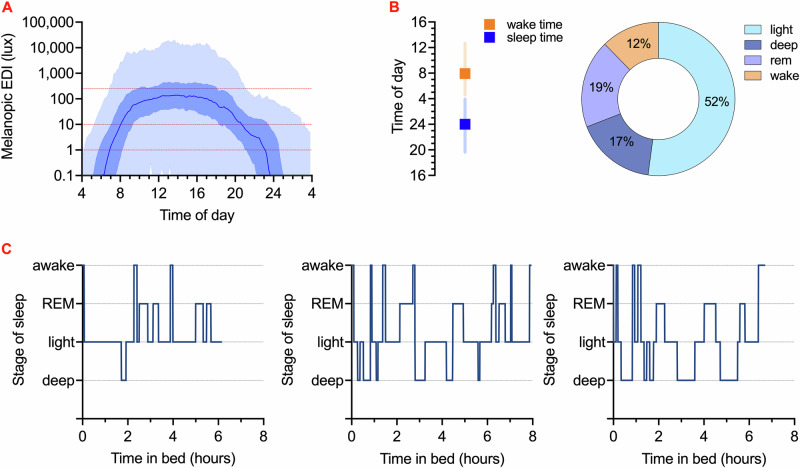
Daily melanopic light exposure and wearable-derived sleep architecture in real-world settings. **A** Melanopic equivalent daylight illuminance (EDI, lux) across the 24-h day. The x-axis represents clock time from 04:00 to 04:00 the following day. The blue line indicates the median, with the first shaded region representing the central 50% of the data and the second shaded region representing 95% of the data. Red dashed lines indicate recommended thresholds: below 1 lux melanopic EDI during sleep, below 10 lux melanopic EDI in the 3 h before sleep, and above 250 lux melanopic EDI during daytime. **B** Left: median sleep onset (blue) and wake time (orange) with minimum–maximum error bars. Right: average proportion of time in bed spent in wake, REM sleep, light sleep, and deep sleep. **C** Representative hypnograms from three days corresponding to minimum, median, and maximum percentages of deep sleep from left to right.

Participants were exposed, on average, to 207.6 min (SD = 141.2) of light exceeding the recommended threshold of 250 lux melanopic EDI per day^31^. However, substantial day-to-day variation was observed, with some days lacking any bright light exposure and others reaching up to 685.5 min of such exposure (Fig. 1A). In comparison to current guidelines^31^, the average light exposure during the 3 h before sleep onset remained within recommended levels, with a mean of 6.6 lux melanopic EDI (SD = 12.1), though 18% of days exceeded the thresholds (10 lux melanopic EDI). Values represent geometric means of melanopic EDI. Similarly, the average lux melanopic EDI during sleep was 0.2 (SD = 0.6), remaining below the 1 lux melanopic EDI recommendation, although some nights still exceeded this level. The last occurrence of 1 lux melanopic EDI exposure often overlapped with sleep onset, indicating a wide range in light exposure duration across participants. The average last exposure to 1000 lux melanopic EDI light—indicative of daylight exposure—occurred at 17:30 (SD = 2.6 h). This measure represents the timing of the final threshold crossing and does not necessarily indicate prolonged or biologically meaningful daylight exposure (see Table S1 for a description of the light exposure metrics).

Light exposure metrics were examined in relation to age, sex, day type (weekdays vs. weekends), photoperiod, and chronotype (mid-sleep time derived from the Munich Chronotype Questionnaire-MCTQ, MSFsc; see Table S2). In our sample, age was not significantly associated with any light exposure metric. Sex differences were observed for interdaily variability (IV), with males having less fragmented exposure patterns. Weekend days were associated with later timing of light exposure, reflected by delayed last exposure above both 1 lux and 1000 lux melanopic EDI thresholds. Chronotype showed consistent associations: participants with later MSFsc exhibited later last exposure above 1 lux melanopic EDI, reduced daytime (M10) and increased nighttime (L5) light exposure, lower interdaily stability (IS), and higher IV, indicating less stable and more fragmented light patterns across study days. Photoperiod demonstrated strong relationships with multiple light metrics, such that longer daylight duration was associated with later last bright light exposure ( > 1000 lux melanopic EDI), later overall light exposure ( > 1 lux melanopic EDI), greater duration of melanopic light exposure ( > 250 lux melanopic EDI), and higher M10 and L5 values. These findings confirm that several wearable-derived light exposure measures are inherently season-dependent and systematically linked to behavioral timing and chronotype.

### Describing natural sleep patterns in real-world contexts

Wearable sleep metrics included a mean sleep duration of 414.3 min (range: 244.0–671.0, SD = 63.7) and an average sleep efficiency of 87.7% (range: 56.6–96.1, SD = 3.4) (see Table S3 for a description of the sleep variables). Participants generally reported no or mild sleep problems. The mean Pittsburgh Sleep Quality Index (PSQI) score was 6.3 (SD = 1.9; *N* = 55) on a scale ranging from 0 to 21. The mean PROMIS Sleep Disturbance score was 18.9 (SD = 6.2; *N* = 34), and the mean PROMIS Sleep Impairment score was 16.7 (SD = 4.2), both on scales ranging from 8 to 40. On average, individuals fell asleep at 00:13 (SD = 1.4 h) and woke at 08:05 (SD = 1.4 h) (Fig. 1B). Chronotype distribution was well represented, with an average MCTQ-derived mid-sleep time (MSFsc) of 4.7 AM (range: 2.3–7.9, SD = 1.3). The average duration of individual deep sleep epochs was 21.9 min (SD = 8.8). Participants typically entered their first REM sleep stage after approximately 2 h (mean = 129.1 min, SD = 66.1). Individuals spent more deep sleep during the first third of sleep. When calculated as a proportion of total sleep duration (excluding wake), participants spent on average 19% in deep sleep, 21% in REM sleep, and 60% in light sleep. Participants were awake for about 1 h each night on average (mean = 58.2 min, SD = 17.0, 12% of the sleep epoch was counted as wake after sleep onset). Substantial inter-individual and day-to-day variability in sleep architecture was observed. For example, the percentage of deep sleep ranged from 3.6% to 39.0%, while REM sleep ranged from 4.8% to 42.3% across all recorded nights (Fig. 1C).

Wearable sleep variables were examined in relation to age, sex, day type (weekdays vs. weekends), photoperiod, and chronotype (see Table S4). Chronotype demonstrated strong associations with key sleep timing parameters, such that later MSFsc was associated with both later sleep onset and later wake times. Day type effects were also evident, with weekend days characterized by delayed sleep onset and wake times compared to weekdays. Sex differences emerged for sleep characteristics, with males exhibiting lower sleep efficiency and shorter sleep duration. The sleep parameters were not significantly associated with either age or photoperiod.

### Associations between light exposure and sleep

**Fig. 2:**
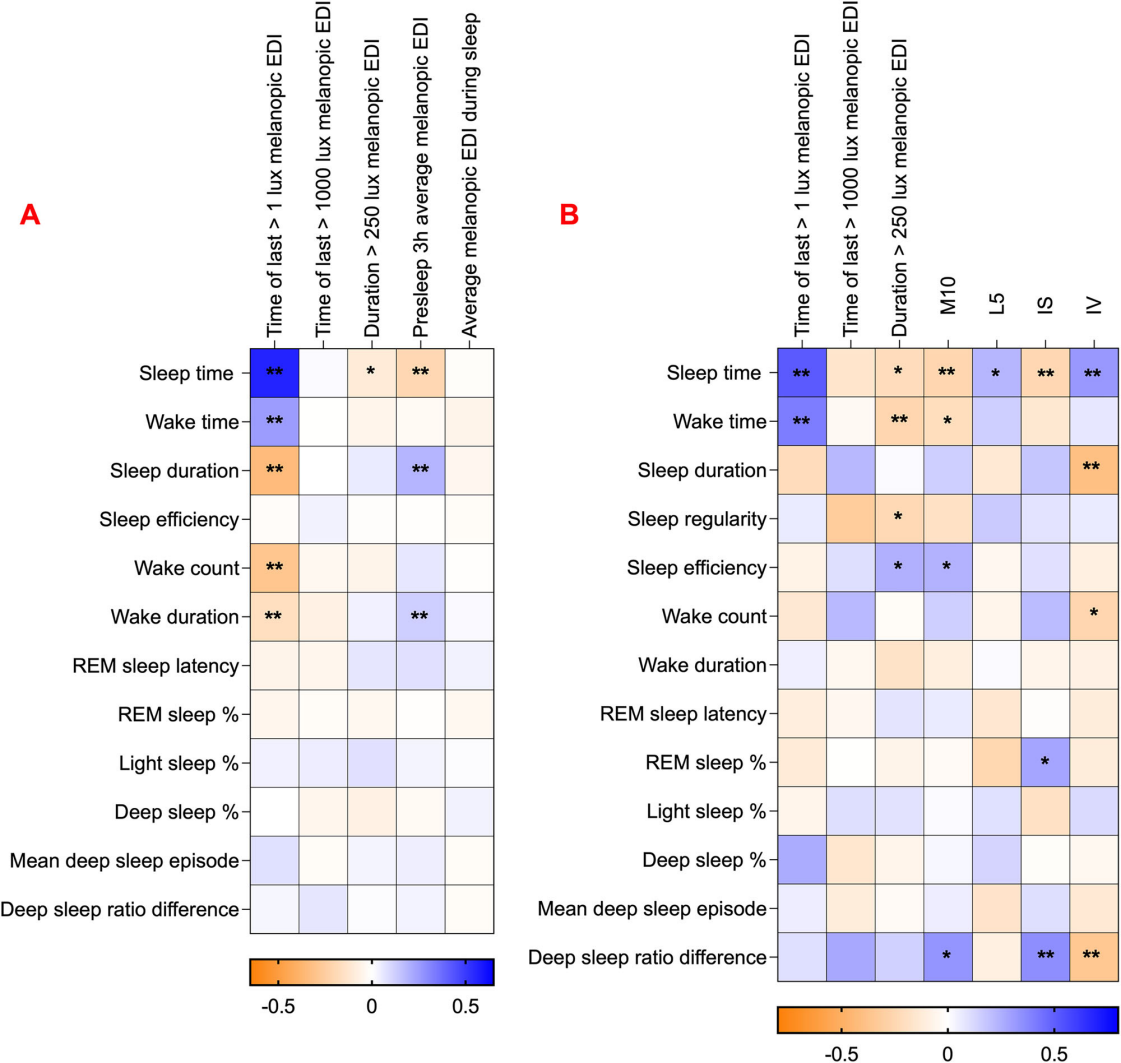
Associations between light exposure metrics and wearable-derived sleep parameters. Associations between **A** daily and **B** weekly light exposure metrics and sleep measured with a Fitbit sleep tracker. Heatmaps show standardized coefficients from separate models: **A** linear mixed model and **B** linear regression. *p < 0.05; **adjusted *p* < 0.05 (Benjamini–Hochberg FDR). *Sleep variables*: Sleep onset time (decimal hour); Wake time (decimal hour); Sleep duration (min); Sleep regularity index (%); Sleep efficiency (%); Wake count (number of night awakenings); Wake duration (min, total duration of night awakenings); REM sleep latency (min, time from sleep onset to first REM episode); REM sleep (% of sleep duration); Light sleep (% of sleep duration); Deep sleep (% of sleep duration); Mean deep sleep episode duration (min); Deep sleep ratio difference (difference in the proportion of deep sleep between the first and last third of sleep). *Light exposure metrics*: Time of last exposure >1 lux melanopic EDI (decimal hour); Time of last exposure >1000 lux melanopic EDI (decimal hour); Duration above 250 lux melanopic EDI (min); Mean light exposure 3 h before sleep (lux melanopic EDI); Average light exposure during the sleep (lux melanopic EDI); M10 (log lux melanopic EDI, mean light exposure during the 10 brightest consecutive hours); L5 (log lux melanopic EDI, mean light exposure during the 5 dimmest consecutive hours); IS (interdaily stability); IV (intradaily variability).

Comparing daily light exposure variables with sleep parameters on a day-by-day basis (Fig. 2A and Table S5), later light exposure and later sleep onset time can be trivially related, as individuals typically reduce or turn off light at bedtimes. This coupling may, in turn, be associated with later wake times, shorter sleep duration, and fewer or shorter nocturnal awakenings. Another noteworthy light exposure variable was the mean melanopic EDI during the 3 h preceding bedtime, which, somewhat unexpectedly, was correlated with earlier sleep onset, longer sleep duration and longer nocturnal awakenings. This association likely reflects a confounding effect of the greater availability of natural daylight in the 3 h pre-sleep in those with earlier chronotypes with early sleep onset time.

We then compared weekly aggregated sleep metrics with light exposure patterns across the study week (Fig. 2B and Table S6). In general, individuals with earlier habitual sleep times were characterized by higher daytime (M10), greater stability of light patterns across days (IS), and less fragmentation within days (IV). Longer durations of exposure above the recommended threshold of 250 lux melanopic EDI were associated with earlier habitual wake times. Longer sleep duration was associated with more consolidated and less fragmented light exposure patterns (IV; *β* = −191.1, SE = 51.2, *p* < 0.001). Notably, individuals exhibiting more stable light exposure (*β* = 0.42, SE = 0.13, *p* = 0.002), less fragmented light exposure (*β* = −0.52, SE = 0.16, *p* < 0.001), and higher daytime light exposure (M10; *β* = 0.11, SE = 0.04, *p* = 0.006) across the week had a higher proportion of deep sleep during the first third of the sleep episode.

### Discrepancies between subjective and wearable-derived sleep measures

**Fig. 3:**
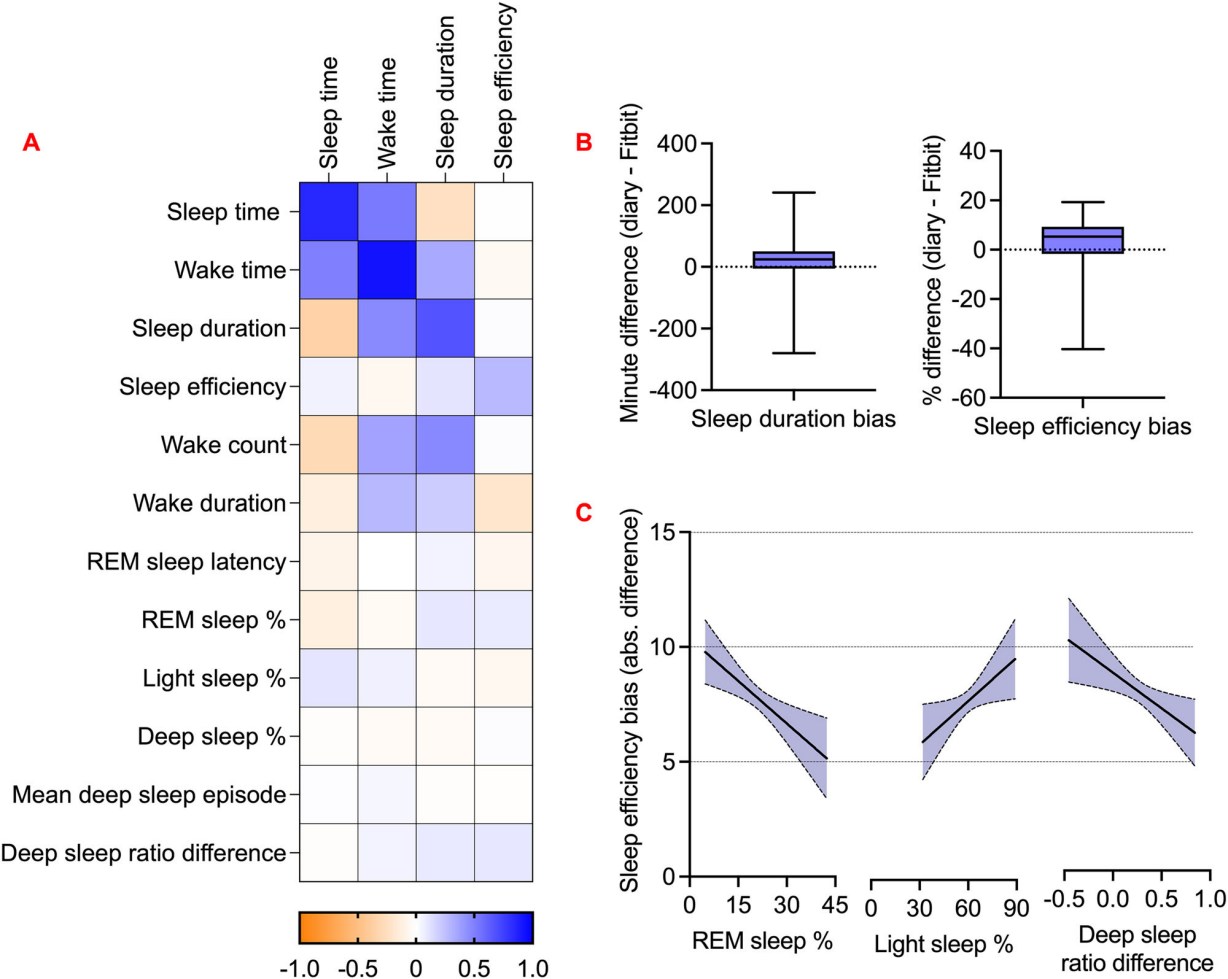
Associations and discrepancies between subjective and wearable-derived sleep measures. **A** Associations between wearable sleep measures (Fitbit sleep tracker) and subjective sleep measures (sleep diary). Associations were tested using linear mixed models with random intercepts for individuals. Heatmaps show standardized coefficients; non-significant associations (adjusted *p* > 0.05, Benjamini–Hochberg FDR) are crossed out. **B** Boxplots of discrepancy between subjective and wearable sleep measures: left, sleep duration (min); right, sleep efficiency (%). Bias was calculated as the difference between subjective and wearable sleep measures (subjective–wearable). Boxplots display the median, interquartile range, and whiskers extending to the minimum and maximum values. **C** Associations between sleep efficiency bias (absolute difference between subjective and wearable sleep measures) and wearable sleep architecture variables: REM sleep (%), light sleep (%), and deep sleep distribution (difference in deep sleep proportion between the first and last third of the night). Linear regression fit lines with 95% confidence intervals are shown.

We also collected daily subjective sleep diaries (N = 528 days). When nested within individuals, correlations between subjective and wearable sleep measures (Fig. 3A and Table S7) revealed strong associations for wake times (*β* = 0.95, SE = 0.02, *p* < 0.001) and sleep onset times (*β* = 0.84, SE = 0.03, *p* < 0.001), with somewhat weaker correlations observed for sleep duration (*β* = 0.65, SE = 0.03, *p* < 0.001) and sleep efficiency (*β* = 0.10, SE = 0.02, *p* < 0.001). While not all variables were available for direct one-to-one comparison, several notable trends emerged, such as increased numbers and durations of nighttime awakenings being associated with longer time spent in bed. Additionally, those with later reported sleep onset times tended to experience reduced REM sleep (*β* = −0.57, SE = 0.23, *p* = 0.015). In a supplementary analysis, we further demonstrated precision between wearable-derived and EEG-based sleep estimates, reinforcing the validity of wrist-worn wearables as objective sleep monitoring tools (Fig. S2).

Given that subjective and wearable sleep measures were not perfectly aligned, we sought to explore the potential sources of this discrepancy. We calculated the absolute differences between subjective and wearable sleep duration and efficiency. Overall, individuals subjectively estimated longer sleep duration and higher sleep efficiency compared to wearable measurements (Fig. 3B). When comparing these discrepancy metrics with wearable sleep characteristics (Fig. 3Cand Table S8), we found that higher sleep efficiency, shorter night wakes, greater deep sleep (particularly in the initial sleep phase), more REM sleep, and lower proportions of light sleep were all associated with reduced discrepancy between subjective and wearable-derived sleep measures. These findings may suggest that higher overall sleep quality is linked to more accurate self-perception of sleep. We then examined the relationship between these discrepancy metrics and both daily and weekly light exposure patterns (Table S9). No significant associations were found.

## Discussion

There is substantial laboratory evidence demonstrating that light influences human sleep timing, quality, and architecture^7,18^. Emerging validation studies have begun to show that light exposure also affects sleep as subjectively reported in everyday life^32^. Building on this foundation, the next step was to collect real-world data to examine the relationship between light exposure and wearable-measured sleep in naturalistic settings. This study implemented a wearable-based approach, combining light dosimetry—capable of capturing melanopic irradiance—with a consumer-grade sleep tracker. These devices were worn simultaneously over multiple days, accompanied by daily self-reported sleep diaries. The large volume of data spanning over 500 days and high participant adherence demonstrated the feasibility of such protocols for future studies. The dataset collected from our study population under real-world conditions revealed associations between sleep characteristics and light exposure patterns, whereby earlier sleep/wake was linked to higher and longer-duration daytime light exposure, and more regular, less fragmented light patterns. The findings highlight that higher interdaily stability and lower intradaily variability of light exposure were associated with greater deep sleep intensity during the initial portion of sleep. Finally, while subjective and wearable sleep measures were generally well correlated, individuals with poorer sleep architecture tended to exhibit higher levels of discrepancies between subjective and wearable-derived sleep measures.

In modern societies, individuals spend a significant portion of their lives indoors, resulting in limited exposure to natural sunlight. Indoor lighting is typically an order of magnitude dimmer than outdoor light and often fails to meet the recommended daytime melanopic EDI levels of at least 250 lux^31^. Conversely, many individuals are overexposed to electric light during the evening and sleep^32^. This pattern of insufficient daytime and excessive nighttime light has been linked to a range of chronic health conditions and increased mortality risk^34,35^. A substantial body of controlled experimental research has demonstrated how light can disrupt circadian rhythms and sleep^7,18^. Additionally, limited time spent outdoors, exposure to nighttime light pollution, and frequent use of screens have all been shown to negatively affect sleep^36–38^. Accordingly, the combination of harmful light exposure patterns and light-induced sleep disruption represents a significant public health concern. Understanding these associations in the context of everyday life is essential for informing strategies that promote healthy lighting behaviors and guide the development of light-based therapeutic interventions. Our study represents a key initial step toward this goal, providing evidence that recommended light exposure patterns are associated with wearable-measured improvements in sleep, even under real-world conditions.

Real-world studies in sleep science offer valuable insights but are inherently limited by their inability to establish causation. Preferred sleep timing is shaped by a combination of endogenous physiological mechanisms and exogenous factors such as light exposure patterns^39^. However, light exposure is not a purely external environmental factor to which individuals are passively subjected; it also covaries with behavioral choices and sleep preferences^23^. This reciprocal relationship is evident in our results; rather than indicating causation, they may reflect that individuals with poor sleep tend to go to bed and wake up later, thereby receiving less and later light exposure. This raises important questions about which light exposure metrics are most appropriate for use in real-world studies. Many of our light exposure metrics were based on sleep timing, such as the average light level during the 3 h before bedtime. However, such measures often produce inconsistent findings, as they may be highly influenced by chronotype and seasonal daylight variations and tend to show elevated values in earlier sleepers. Therefore, the interpretation of these results should be approached with caution. Future studies should aim to refine or develop more robust metrics that capture pre-sleep light exposure independently of individual sleep timing, to better isolate the true effects of light on sleep outcomes.

Previous real-world studies using melanopic EDI have shown that higher pre-bedtime light exposure is associated with longer sleep onset latency, while earlier sleep timing correlates with more robust and reproducible daily light exposure patterns, characterized by higher daytime and lower nighttime light levels^32^. However, that study lacked objective sleep staging data. To address this gap, we examined both light exposure and sleep architecture and found that high interdaily stability and low intradaily variability were associated with a greater proportion of deep sleep concentrated in the early phases of the night. To our knowledge, this is the first report linking stability in light exposure patterns with NREM sleep distribution. A controlled laboratory study demonstrated that bright morning light can advance the circadian phase without affecting NREM sleep homeostasis^20^. However, complementary field studies using actigraphy and subsequent polysomnography have further shown that higher morning light levels and earlier first light exposure are associated with increased slow-wave sleep percentage^19^. Similarly, another study reported that higher midday light exposure was associated with longer REM sleep latency and a shift of REM sleep toward the later portion of the night^40^. The circadian pacemaker and sleep homeostat interact to regulate consolidated wake and sleep periods across the day-night cycle^6^. Overall, morning daylight exposure may enhance the amplitude and stability of circadian rhythms through SCN-mediated photic entrainment^41^. This strengthened circadian signaling, potentially accompanied by a phase advance that more effectively gates sleep homeostasis, together with increased homeostatic sleep pressure—arising either from direct effects of light or indirectly via greater physical activity and behavioral activation associated with higher light exposure—may promote greater slow-wave activity during the initial sleep cycles^11,42,43^.

Subjective sleep measures are widely used in epidemiological research and often show concordance with objective assessments; however, inconsistencies between subjective and objective sleep estimates are also documented^44–46^. Our protocol, which simultaneously collected subjective and wearable-derived sleep data, enabled the investigation of these discrepancies. Notably, wearable and subjective sleep parameters were strongly correlated in our study. Consumer-grade wearables are not gold-standard objective measures and cannot be assumed to be superior to subjective reports, which are also prone to reporting bias. Sleep misperception, frequently described in individuals with insomnia and depression, who tend to underreport their sleep quality^47,48^. While previous research typically compares subjective surveys to single-night polysomnography, our approach provides ecologically valid, longitudinal insights. We observed that a higher proportion of light sleep, lower REM sleep, and reduced deep sleep during the first third of the night were associated with greater discrepancies between subjective and wearable sleep measures. One explanation is that dissatisfaction and dysfunctional beliefs may bias subjective sleep perception^49,50^. Alternatively, reductions in REM and slow-wave sleep—stages critical for memory consolidation—may impair the accuracy of sleep recall^51–53^. Although measurement error in wearable sleep tracking remains a consideration^54,55^, the magnitude of discrepancy between subjective and wearable sleep measures exceeded the expected device error, suggesting that these discrepancies may reflect meaningful psychological or neurophysiological mechanisms, even in healthy adults.

Since light exerts its physiological effects primarily through the photopigment melanopsin, a melanopic-weighted metric provides a more accurate approach for studying non-visual responses to light^13,14,30,56^. Melanopic irradiance has been shown to predict light’s ability to suppress evening melatonin, delay melatonin onset, decrease sleep latency, and reduce slow wave activity^57–59^. Monitoring light exposure using melanopic EDI is a key strength of our study. However, several limitations should be acknowledged. First, light was measured at the wrist, whereas the non-visual effects of light depend on retinal exposure, and the accuracy of wrist-based monitoring for this purpose remains unvalidated. As such, wrist-worn sensors may not capture the directionality of incident light reaching the eyes and can underestimate exposure from near-field, gaze-dependent sources such as smartphones, tablets, and televisions, particularly in the evening when ambient levels are low. Together, these limitations mean that our conclusions regarding compliance with recommended evening and nighttime light thresholds should be interpreted cautiously. Additionally, the light sensors used in this study are underpowered to detect very dim nighttime light levels below 1 lux melanopic EDI, potentially affecting the detection of subtle circadian influences. Another limitation concerns the wearable sleep measurements, which, although practical for real-world use, still have a margin of error compared to gold-standard polysomnography^54,55,60^. Real-world studies also face the challenge of substantial interindividual variability in light sensitivity, which may obscure subtle effects^61^. The study also did not control for physical activity, meal timing, or other secondary zeitgebers that may affect sleep. Given the relatively small effect sizes observed, future research would benefit from larger, multicultural samples with broad variation in age, health status, and demographic backgrounds to improve generalizability and statistical power.

## Methods

### Participants

Data were drawn from two cohorts of healthy adults residing in Manchester, UK. The first cohort (*n* = 60) was recruited between July 2022 and August 2023, and the second cohort (*n* = 51) was enrolled between December 2023 and September 2024. The study aimed to recruit a demographically diverse sample to reflect natural variation in the general population under real-world conditions. In total, data from 111 individuals were initially collected. 22 participants were excluded from the final analysis due to insufficient data (i.e., fewer than two days of valid light and sleep recordings), resulting in a final analytic sample of 89 participants. Participants were eligible for inclusion if they were at least 18 years old, had not undertaken intercontinental travel in the preceding two weeks, were not engaged in night shift work, and had no diagnosed sleep disorders. In addition, cohort-specific exclusion criteria were applied: students were excluded from the first cohort, while individuals with a current diagnosis of depression were excluded from the second. The study was approved by the University of Manchester Research Ethics Committee (Ref: 2021-12948-20856, 2023-16080-26819, and 2023-18265-31575). All participants provided written informed consent prior to participation.

The two cohorts were originally established as independent studies designed to investigate distinct primary hypotheses (see Table S10 for sample descriptions). In the first cohort, students were excluded, and the primary objective was to examine associations between light exposure and cognitive performance in employed adults. The second cohort excluded individuals with a current diagnosis of depression and was designed to investigate associations between light exposure and mood in non-depressed adults. Despite these differing primary aims, both cohorts were harmonized from the outset to address shared hypotheses concerning melanopic light exposure and objectively measured sleep characteristics. Accordingly, monitoring procedures, instrumentation, and recruitment protocols were standardized across cohorts. Each cohort was designed to recruit a minimum of 30 participants, consistent with prior power considerations for personal melanopic light exposure studies conducted under real-world conditions^62^. As the study aimed to capture naturalistic, real-world behavior, we avoided overly restrictive health-related exclusion criteria. All participants were considered generally healthy, with the exception of a small number reporting a history of ADHD (*n* = 4) or migraine (*n* = 5). A subset of participants reported a prior history of depression or anxiety (*n* = 14); however, none had an active diagnosis or were receiving medication during the study period.

### Study procedure

A real-world protocol was employed to monitor participants’ personal light exposure and sleep patterns^25,32^. Each participant was enrolled in the study for a minimum of one week, with the option to withdraw at any time or extend their participation beyond the initial study period. Study weeks commenced on any weekday, depending on individual scheduling availability. Data collection involved the use of two wearable devices: (1) a wrist-worn light sensor and (2) a wristband sleep tracker, Fitbit Charge 5 (Fitbit Inc., San Francisco, CA, USA). Participants also received links to complete daily and baseline surveys via the Qualtrics online platform (Qualtrics, Provo, UT). At the initial onboarding session, participants were provided with the devices and given detailed instructions on proper usage. Light loggers and Fitbit devices were synchronized with the experimenter’s local computer clock and activated during the onboarding session. They then completed a baseline survey. Throughout the study period, participants were instructed to wear the light sensor during all waking hours and the sleep tracker continuously, including during sleep. Each morning upon waking, participants completed a brief online sleep diary. For light monitoring, participants were asked to remove the device just before going to bed and place it in a consistent location within their sleeping environment—preferably near eye level—to continue recording ambient nighttime light exposure. They were also advised to avoid wearing long-sleeved clothing that might block the light sensor. We selected wrist-worn monitoring because it represents the least invasive approach and minimizes interference with participants’ everyday behaviors, which was essential given our primary aim of capturing naturalistic, real-world light exposure. To ensure ecologically valid data, participants were explicitly encouraged to maintain their normal daily routines and behaviors without altering their lifestyle for the study.

### Surveys

At baseline, participants completed a study-specific sociodemographic and health questionnaire, as well as standardized assessments of sleep and chronotype. All participants completed the Munich Chronotype Questionnaire (MCTQ) to assess circadian preferences^63^. In addition, participants in the first cohort completed the Pittsburgh Sleep Quality Index (PSQI)^64^, while those in the second cohort completed the PROMIS Sleep Disturbance (v. 1.0; 8b) and PROMIS Sleep-Related Impairment (v. 1.0; 8a) scales to evaluate sleep quality and daytime functioning^65^. The sociodemographic and health survey captured information on age, sex, employment status, shift work involvement, self-rated health, and any history of sleep, visual, psychiatric, or neurological disorders. Each morning during the study period, participants completed a brief online sleep diary. The diary recorded self-reported bedtime and wake time, total sleep duration (in minutes) and sleep onset latency (the time taken to fall asleep, in minutes).

### Light and sleep monitoring devices

Ambient light exposure was recorded using wrist-worn light sensors capable of measuring melanopic EDI with precise timestamps. Devices were worn on the non-dominant wrist throughout the waking period. In the first cohort, participants used the Spectrawear device (University of Manchester)^33^, configured to record light data at 30-s intervals. Due to limited battery life, participants were instructed to charge the device daily, preferably overnight. Participants in the second cohort were equipped with the ActLumus Condor light sensor (Condor Instruments, Brazil), which continuously sampled environmental light at 1-min intervals. As these devices are not waterproof, participants were instructed to remove them during activities involving water exposure—such as heavy rain, showering, or swimming—and, where possible, to store the device in a comparable lighting environment during removal periods. This device’s battery capacity exceeded the study duration, so chargers were not provided.

All participants were also fitted with a Fitbit Charge 5 (Fitbit Inc., San Francisco, CA, USA) worn on the dominant wrist to measure sleep. Devices were activated during the onboarding session using a researcher-controlled phone and Fitbit account. Participants were blinded to their sleep data during the study week, and charging was not required. Prior to each deployment, devices were reset and set up with participant-specific data (age, sex, height, and weight) to improve sleep estimation accuracy. The sensitive sleep detection mode was enabled, and devices were configured in battery-saving and Do Not Disturb modes to prevent screen interactions. Participants were instructed not to modify any device settings throughout the study.

### Light exposure metrics

All melanopic EDI (lux) values were log_10_-transformed prior to analysis to normalize their distribution. Each study day was defined as the 24-h period from 04:00 to 04:00, a window selected to capture the majority of participants’ bedtimes within a single day. Light exposure metrics were calculated separately for daily and weekly timescales. Only complete days—defined as those with no more than 30 min of missing light data—were included in the analysis (*N* = 542 days). Light exposure data were subsequently aligned with the following nocturnal sleep episode to examine associations between light and sleep outcomes (mean number of matched days per participant was 5.2 days, *N* = 454). Light metrics were derived using the LightLogR package and scripts, which were employed to extract and compute relevant variables from the raw time-stamped melanopic EDI data^66^.

Light exposure metrics were selected to represent key biologically relevant dimensions, including timing, level, duration, and the stability and fragmentation of light patterns. Recommended light exposure thresholds^31^ informed the derivation of these variables. Daily light exposure metrics were calculated, including: total time above 250 lux melanopic EDI (TAT250, in minutes); clock time of the last daily occurrence of ≥1000 lux melanopic EDI; clock time of the last daily occurrence of ≥1 lux melanopic EDI; average light level during the 3 h preceding sleep onset (lux melanopic EDI); and average light level between sleep onset and final awakening (lux melanopic EDI). Using the full week of data, the following variables were extracted: weekly averages of TAT250, 1000 lux melanopic EDI last time, and 1 lux melanopic EDI last time, as well as M10 (mean light level during the brightest 10-h period; log lux melanopic EDI), L5 (mean light level during the dimmest 5-h period; log lux melanopic EDI), IS (interdaily stability of light exposure), and IV (intradaily variability of light exposure). IS quantifies the degree of day-to-day regularity by comparing the variance of the hourly mean light profile to the overall variance of the time series. Higher IS values indicate greater stability of light exposure patterns across days. IV measures the fragmentation of the rhythm within a day and was computed from successive differences in the time series relative to the overall variance. Higher IV values reflect more frequent fluctuations and greater within-day fragmentation of light exposure.

### Sleep metrics

The Fitbit estimates sleep parameters using a combination of accelerometer-based movement, heart rate variability, and skin temperature data. The device automatically detects sleep episodes and records sleep data at a 1-min resolution, classifying each epoch into one of four stages: wake, light sleep, deep sleep (slow-wave sleep), and rapid eye movement (REM) sleep. Fitbit data were cleaned by excluding records with sleep onset before 19:00 or after 04:00, wake time before 04:00 or after 13:00, and sleep duration <4 h (*N* = 547). For each sleep episode, the following variables were extracted: bedtime, wake time, total sleep duration (in minutes), sleep efficiency (percentage), number of awakenings, total duration of nocturnal wakefulness between sleep onset and final awakening (in minutes), and the percentage of time spent in each sleep stage (deep, light, REM) relative to total sleep duration. Additional sleep metrics included REM sleep latency (time from sleep onset to the first REM episode, in minutes), the difference in the proportion of deep sleep between the first and last third of the sleep episode, and sleep consolidation, defined as the average duration of deep sleep blocks (in minutes). Modified sleep regularity index (SRI) was calculated as the average percentage of minute-by-minute similarity in sleep–wake state between each pair of consecutive days. To enable comparisons with subjective sleep reports, only days with overlapping sleep diary entries were retained (N = 528). The sleep diary recorded sleep duration (in minutes), sleep and wake times, and sleep efficiency, which was calculated as the percentage of time spent in bed that was actually spent asleep. Sleep onset time was defined as the sum of bedtime and sleep latency. Variables representing bias in perceived sleep duration and efficiency were calculated as the absolute differences between self-reported (subjective) and device-measured values.

### Statistical analysis

All data processing and statistical analyses were performed using R version 4.5.1 (2025). Descriptive statistics were reported as means and standard deviations for continuous variables and as frequencies for categorical variables. Two-tailed *p*-values were used for hypothesis testing, with a statistical significance threshold set at 0.05. Where multiple hypothesis tests were performed, *p*-values were adjusted using the Benjamini–Hochberg procedure to control the false discovery rate (FDR), i.e., the expected proportion of incorrectly rejected null hypotheses among significant results.

Age group ( > 30 vs. <30 years), sex (male vs. female), photoperiod (hours), day type (weekday vs. weekend), and MCTQ-derived mid-sleep time (MSFsc) were included as covariates in the analyses. To investigate associations between objective sleep metrics and covariates, as well as between daily light exposure metrics and covariates, linear mixed-effects models with random intercepts for participants were performed. Separate models were fitted for each outcome variable, including all covariates as predictors. For weekly light exposure metrics, multivariate linear regression models were conducted, again fitting one model per outcome and including all covariates (except day type) as predictors.

Associations between daily light exposure metrics and daily objective sleep parameters, daily light exposure and daily discrepancy between subjective and objective sleep variables, daily objective sleep measures and daily discrepancy between subjective and objective sleep variables were analyzed using linear mixed-effects models with random intercepts for participants, including covariates. Objective and subjective sleep variables were analyzed using linear mixed-effects models with random intercepts. Standardized coefficients were visualized using heatmaps for comparative interpretation. All fixed-effect coefficients were standardized by rescaling both predictors and outcomes by their respective sample standard deviations, enabling the presentation of interpretable standardized coefficients. Analysis of variance (ANOVA) was subsequently performed on the fitted mixed-effects models to assess the statistical significance of fixed effects. Each F-statistic and its degrees of freedom were converted into partial *η*² values as measures of effect size. Analyses utilized the following R packages: lme4 (v1.1-37), lmerTest (v3.1-3), parameters (v0.27.0), and effectsize (v1.0.1).

For weekly-level analyses, objective sleep and discrepancy between subjective and objective sleep metrics were aggregated as person-level means across the full dataset. Associations between weekly light exposure metrics and weekly objective sleep measures, weekly light exposure and weekly discrepancy between subjective and objective sleep metrics were examined using linear regression models, including covariates. Standardized regression coefficients were visualized via heatmaps for comparative analysis, and R² values were reported as effect size estimates.

As a supplementary analysis to assess the correlation between devices, an ActLumus Condor light sensor and Spectrawear were worn side-by-side on the same arm by a participant for a continuous period of 4.5 days (1-min sampling interval, *N* = 6730). Lux melanopic EDI values from both devices were compared using linear regression analysis. Subsequently, a white LED light source was tested across seven intensity levels, ranging from 0 to 4000 lux melanopic EDI, and fluorescent bulbs were tested across eight intensity steps, ranging from 0 to 2500 lux melanopic EDI. Spectral irradiance measurements were obtained using a calibrated SpectroCAL MKII Spectroradiometer (Cambridge Research Systems, UK), and lux melanopic EDI values were again compared using linear regression models. In an additional supplementary analysis, to evaluate the correspondence between sleep metrics, a Fitbit Charge 5 and a Dreem 3 EEG headband (Beacon Biosignals, Boston, MA) were worn simultaneously by two individuals over a total of 30 nights. Sleep parameters obtained from both devices were compared using linear regression models.

## Supplementary information

Supplementary materials-20260316

## Data Availability

Software and hardware designs of the wearable light dosimeter are available in a repository (https:/github.com/Non-Invasive-Bioelectronics-Lab/Wearable_Light_Sensor_Public). R script code to process and analyze the data and anonymized data of light exposure and sleep created for the study are available in a GitHub repository (https://github.com/altugdidikoglu/light-sleep-inreallife).

## References

[1] Chaput, J. P. et al. The role of insufficient sleep and circadian misalignment in obesity. *Nat. Rev. Endocrinol.* **19**, 82–97 (2023). 36280789 10.1038/s41574-022-00747-7 PMC9590398

[2] Tobaldini, E. et al. Sleep, sleep deprivation, autonomic nervous system and cardiovascular diseases. *Neurosci. Biobehav. Rev.* **74**, 321–329 (2017). 27397854 10.1016/j.neubiorev.2016.07.004

[3] Freeman, D., Sheaves, B., Waite, F., Harvey, A. G. & Harrison, P. J. Sleep disturbance and psychiatric disorders. *Lancet Psychiatry* **7**, 628–637 (2020). 32563308 10.1016/S2215-0366(20)30136-X

[4] Zhang, Y. et al. Sleep in Alzheimer’s disease: a systematic review and meta-analysis of polysomnographic findings. *Transl. Psychiatry* **12**, 136 (2022). 35365609 10.1038/s41398-022-01897-y PMC8976015

[5] Chuang, S. C. Sleep habits are associated with cognition decline in physically robust, but not in frail participants: a longitudinal observational study. *Sci. Rep.* **12**, 11595 (2022). 35804185 10.1038/s41598-022-15915-y PMC9270465

[6] Dijk, D. J. & Czeisler, C. A. Paradoxical timing of the circadian rhythm of sleep propensity serves to consolidate sleep and wakefulness in humans. *Neurosci. Lett.* **166**, 63–68 (1994). 8190360 10.1016/0304-3940(94)90841-9

[7] Blume, C., Garbazza, C. & Spitschan, M. Effects of light on human circadian rhythms, sleep and mood. *Somnologie* **23**, 147–156 (2019). 31534436 10.1007/s11818-019-00215-x PMC6751071

[8] Foster, R. G., Hughes, S. & Peirson, S. N. Circadian Photoentrainment in Mice and Humans. *Biology* **9**, 1–45 (2020). 10.3390/biology9070180 PMC7408241 32708259

[9] Lewy, A. J., Wehr, T. A., Goodwin, F. K., Newsome, D. A. & Markey, S. P. Light suppresses melatonin secretion in humans. *Science* **210**, 1267–1269 (1980). 7434030 10.1126/science.7434030

[10] Cajochen, C. Alerting effects of light. *Sleep Med. Rev.* **11**, 453–464 (2007). 17936041 10.1016/j.smrv.2007.07.009

[11] Zhang, Z., Beier, C., Weil, T. & Hattar, S. The retinal ipRGC-preoptic circuit mediates the acute effect of light on sleep. *Nat. Commun.* **12**, 5115 (2021). 34433830 10.1038/s41467-021-25378-w PMC8387462

[12] Zeitzer, J. M., Dijk, D. J., Kronauer, R. E., Brown, E. N. & Czeisler, C. A. Sensitivity of the human circadian pacemaker to nocturnal light: melatonin phase resetting and suppression. *J. Physiol.* **526**, 695–702 (2000). 10922269 10.1111/j.1469-7793.2000.00695.x PMC2270041

[13] Santhi, N. et al. The spectral composition of evening light and individual differences in the suppression of melatonin and delay of sleep in humans. *J. Pineal Res.* **53**, 47–59 (2012). 22017511 10.1111/j.1600-079X.2011.00970.x

[14] Cajochen, C., Stefani, O., Schöllhorn, I., Lang, D. & Chellappa, S. L. Influence of evening light exposure on polysomnographically assessed night-time sleep: A systematic review with meta-analysis. *Light. Res. Technol.* **54**, 609–624 (2022).

[15] Cho, C. H. et al. Exposure to dim artificial light at night increases REM sleep and awakenings in humans. *Chronobiol. Int.* **33**, 117–123 (2016). 26654880 10.3109/07420528.2015.1108980

[16] He, M., Ru, T., Li, S., Li, Y. & Zhou, G. Shine light on sleep: morning bright light improves nocturnal sleep and next morning alertness among college students. *J. Sleep Res.* **32**, e13724 (2023). 36058557 10.1111/jsr.13724

[17] Minors, D. S., Waterhouse, J. M. & Wirz-Justice, A. A human phase-response curve to light. *Neurosci. Lett.* **133**, 36–40 (1991). 1791996 10.1016/0304-3940(91)90051-t

[18] Blume, C. & Münch, M. Effects of light on biological functions and human sleep. *Handb. Clin. Neurol.* **206**, 3–16 (2025). 39864930 10.1016/B978-0-323-90918-1.00008-3

[19] Wams, E. J. et al. Linking light exposure and subsequent sleep: a field polysomnography study in humans. *Sleep* **40**, zsx165 (2017). 29040758 10.1093/sleep/zsx165 PMC5806586

[20] Dijk, D. J., Beersma, D. G. M., Daan, S. & Lewy, A. J. Bright morning light advances the human circadian system without affecting NREM sleep homeostasis. *Am. J. Physiol.* **256**, R106–R111 (1989). 2912203 10.1152/ajpregu.1989.256.1.R106

[21] Figueiro, M. G. et al. The impact of daytime light exposures on sleep and mood in office workers. *Sleep Health* **3**, 204–215 (2017). 28526259 10.1016/j.sleh.2017.03.005

[22] Nagare, R. et al. Access to daylight at home improves circadian alignment, sleep, and mental health in healthy adults: a crossover study. *Int. J. Environ. Res. Public Health* **18**, 9980 (2021). 34639284 10.3390/ijerph18199980 PMC8507741

[23] Biller, A. M., Balakrishnan, P. & Spitschan, M. Behavioural determinants of physiologically-relevant light exposure. *Commun. Psychol.* **2**, 114 (2024). 39614105 10.1038/s44271-024-00159-5 PMC11607403

[24] Biller, A. M. et al. The Ecology of Human Sleep (EcoSleep) Cohort Study: Protocol for a longitudinal repeated measurement burst design study to assess the relationship between sleep determinants and outcomes under real-world conditions across time of year. *J. Sleep Res.* **34**, e14225 (2025). 39039613 10.1111/jsr.14225 PMC11911042

[25] Guidolin, C. et al. Protocol for a prospective, multicentre, cross-sectional cohort study to assess personal light exposure. *BMC Public Health* **24**, 3285 (2024). 39592960 10.1186/s12889-024-20206-4 PMC11590290

[26] Mead, M. P., Reid, K. J. & Knutson, K. L. Night-to-night associations between light exposure and sleep health. *J. Sleep Res.* **32**, e13620 (2023). 35599235 10.1111/jsr.13620 PMC9679040

[27] Mitsui, K. et al. Short-wavelength light exposure at night and sleep disturbances accompanied by decreased melatonin secretion in real-life settings: a cross-sectional study of the HEIJO-KYO cohort. *Sleep Med.* **90**, 192–198 (2022). 35190318 10.1016/j.sleep.2022.01.023

[28] Esaki, Y. et al. Interaction of daytime and nighttime light exposure on objective sleep quality in patients with bipolar disorder: a cross-sectional analysis of the APPLE cohort. *Transl. Psychiatry* **15**, 291 (2025). 40825765 10.1038/s41398-025-03549-3 PMC12361580

[29] Obayashi, K. et al. Daily light exposure profiles and the association with objective sleep quality in patients with Parkinson’s disease: the PHASE study. *Sleep* **47**, zsae036 (2024). 38330229 10.1093/sleep/zsae036 PMC11321845

[30] Lucas, R. J. et al. Measuring and using light in the melanopsin age. *Trends Neurosci.* **37**, 1–9 (2014). 24287308 10.1016/j.tins.2013.10.004 PMC4699304

[31] Brown, T. M. et al. Recommendations for daytime, evening, and nighttime indoor light exposure to best support physiology, sleep, and wakefulness in healthy adults. *PLoS Biol.* **20**, e3001571 (2022). 35298459 10.1371/journal.pbio.3001571 PMC8929548

[32] Didikoglu, A. et al. Associations between light exposure and sleep timing and sleepiness while awake in a sample of UK adults in everyday life. *Proc. Natl. Acad. Sci. USA* **120**, e2301608120 (2023). 37812713 10.1073/pnas.2301608120 PMC10589638

[33] Mohammadian, N. et al. A wrist-worn internet of things sensor node for wearable equivalent daylight illuminance monitoring. *IEEE Internet Things J* **11**, 16148 (2024). 38765485 10.1109/JIOT.2024.3355330 PMC11100858

[34] Windred, D. P. et al. Brighter nights and darker days predict higher mortality risk: a prospective analysis of personal light exposure in >88,000 individuals. *Proc Natl. Acad. Sci. USA* **121**, e2405924121 (2024). 39405349 10.1073/pnas.2405924121 PMC11513964

[35] Mason, I. C. et al. Light exposure during sleep impairs cardiometabolic function. *Proc. Natl. Acad. Sci. USA* **119**, e2113290119 (2022). 35286195 10.1073/pnas.2113290119 PMC8944904

[36] Burns, A. C. et al. Time spent in outdoor light is associated with mood, sleep, and circadian rhythm-related outcomes: A cross-sectional and longitudinal study in over 400,000 UK Biobank participants. *J. Affect. Disord.* **295**, 347–352 (2021). 34488088 10.1016/j.jad.2021.08.056 PMC8892387

[37] Chang, A. M., Aeschbach, D., Duffy, J. F. & Czeisler, C. A. Evening use of light-emitting eReaders negatively affects sleep, circadian timing, and next-morning alertness. *Proc. Natl. Acad. Sci. USA* **112**, 1232–1237 (2015). 25535358 10.1073/pnas.1418490112 PMC4313820

[38] Paksarian, D. et al. Association of Outdoor Artificial Light at Night With Mental Disorders and Sleep Patterns Among US Adolescents. *JAMA Psychiatry* **77**, 1266–1275 (2020). 32639562 10.1001/jamapsychiatry.2020.1935 PMC7344797

[39] Skeldon, A. C. et al. Method to determine whether sleep phenotypes are driven by endogenous circadian rhythms or environmental light by combining longitudinal data and personalised mathematical models. *PLoS Comput. Biol.* **19**, e1011743 (2023). 38134229 10.1371/journal.pcbi.1011743 PMC10817199

[40] Nowozin, C. et al. Living in Biological Darkness II: Impact of Winter Habitual Daytime Light on Night-Time Sleep. *Eur. J. Neurosci.* **61**, e16647 (2025). 39831471 10.1111/ejn.16647 PMC11744753

[41] Bano-Otalora, B. et al. Bright daytime light enhances circadian amplitude in a diurnal mammal. *Proc. Natl. Acad. Sci. USA* **118**, e2100094118 (2021). 34031246 10.1073/pnas.2100094118 PMC8179182

[42] Tsai, J. W. et al. Melanopsin as a sleep modulator: circadian gating of the direct effects of light on sleep and altered sleep homeostasis in Opn4(-/-) mice. *PLoS Biol.* **7**, e1000125 (2009). 19513122 10.1371/journal.pbio.1000125 PMC2688840

[43] Cajochen, C. et al. Evidence that homeostatic sleep regulation depends on ambient lighting conditions during wakefulness. *Clocks Sleep* **1**, 517–531 (2019). 33089184 10.3390/clockssleep1040040 PMC7445844

[44] Masaki, M. et al. Discrepancies between subjective and objective sleep assessments revealed by in-home electroencephalography during real-world sleep. *Proc. Natl. Acad. Sci. USA* **122**, e2412895121 (2025). 39819218 10.1073/pnas.2412895121 PMC11761674

[45] Pierson-Bartel, R. & Ujma, P. P. Objective sleep quality predicts subjective sleep ratings. *Sci. Rep.* **14**, 5943 (2024). 38467694 10.1038/s41598-024-56668-0 PMC10928218

[46] Armitage, R., Trivedi, M., Hoffmann, R. & Rush, A. Relationship between objective and subjective sleep measures in depressed patients and healthy controls. *Depress. Anxiety* **5**, 97–102 (1997). 9262940 10.1002/(sici)1520-6394(1997)5:2<97::aid-da6>3.0.co;2-2

[47] Carney, A. E., Wescott, D. L., Carmona, N. E., Carney, C. E. & Roecklein, K. A. The role of beliefs about sleep in nightly perceptions of sleep quality across a depression continuum. *J. Affect. Disord.* **311**, 440 (2022). 35597468 10.1016/j.jad.2022.05.092 PMC9523734

[48] Harvey, A. G. & Tang, N. K. Y. Misperception of sleep in insomnia: a puzzle and a resolution. *Psychol. Bull.* **138**, 77–101 (2012). 21967449 10.1037/a0025730 PMC3277880

[49] Blake, M. J., Trinder, J. A. & Allen, N. B. Mechanisms underlying the association between insomnia, anxiety, and depression in adolescence: Implications for behavioral sleep interventions. *Clin. Psychol. Rev.* **63**, 25–40 (2018). 29879564 10.1016/j.cpr.2018.05.006

[50] Ohayon, M. M. & Milesi, C. Artificial Outdoor Nighttime Lights Associate with Altered Sleep Behavior in the American General Population. *Sleep* **39**, 1311–1320 (2016). 27091523 10.5665/sleep.5860 PMC4863221

[51] Kong, J., Zhou, L., Li, X. & Ren, Q. Sleep disorders affect cognitive function in adults: an overview of systematic reviews and meta-analyses. *Sleep Biol. Rhythms* **21**, 133–142 (2023). 38469285 10.1007/s41105-022-00439-9 PMC10900040

[52] Casey, S. J. et al. Slow wave and REM sleep deprivation effects on explicit and implicit memory during sleep. *Neuropsychology* **30**, 931–945 (2016). 27797541 10.1037/neu0000314

[53] Brunet, J. F., McNeil, J., Doucet, É. & Forest, G. The association between REM sleep and decision-making: supporting evidences. *Physiol. Behav.* **225**, 113109 (2020). 32730842 10.1016/j.physbeh.2020.113109

[54] Kim, K., Park, D. Y., Song, Y. J., Han, S. & Kim, H. J. Consumer-grade sleep trackers are still not up to par compared to polysomnography. *Sleep Breath* **26**, 1573–1582 (2022). 34741243 10.1007/s11325-021-02493-y

[55] Park, J.-E., Ahn, E. K., Yoon, K. & Kim, J. Performance of Fitbit devices as tools for assessing sleep patterns and associated factors. *J. Sleep Med.* **21**, 59–64 (2024).

[56] Brown, T. M. Melanopic illuminance defines the magnitude of human circadian light responses under a wide range of conditions. *J. Pineal Res.* **69**, e12655 (2020). 32248548 10.1111/jpi.12655

[57] Schöllhorn, I. et al. Melanopic irradiance defines the impact of evening display light on sleep latency, melatonin and alertness. *Commun. Biol.* **6**, 228 (2023). 36854795 10.1038/s42003-023-04598-4 PMC9974389

[58] Chellappa, S. L. et al. Acute exposure to evening blue-enriched light impacts on human sleep. *J. Sleep Res.* **22**, 573–580 (2013). 23509952 10.1111/jsr.12050

[59] Münch, M. et al. Wavelength-dependent effects of evening light exposure on sleep architecture and sleep EEG power density in men. *Am. J. Physiol. Regul. Integr. Comp. Physiol.* **290**, R1421–R1428 (2006). 16439671 10.1152/ajpregu.00478.2005

[60] Lee, Y. J., Lee, J. Y., Cho, J. H., Kang, Y. J. & Choi, J. H. Performance of consumer wrist-worn sleep tracking devices compared to polysomnography: a meta-analysis. *J. Clin. Sleep Med.* **21**, 573–582 (2025). 39484805 10.5664/jcsm.11460 PMC11874098

[61] Phillips, A. J. K. et al. High sensitivity and interindividual variability in the response of the human circadian system to evening light. *Proc. Natl. Acad. Sci. USA* **116**, 12019–12024 (2019). 31138694 10.1073/pnas.1901824116 PMC6575863

[62] Zauner, J., Udovicic, L. & Spitschan, M. Power analysis for personal light exposure measurements and interventions. *PLoS One* **19**, e0308768 (2024). 39661605 10.1371/journal.pone.0308768 PMC11633969

[63] Roenneberg, T., Wirz-Justice, A. & Merrow, M. Life between clocks: daily temporal patterns of human chronotypes. *J. Biol. Rhythms* **18**, 80–90 (2003). 12568247 10.1177/0748730402239679

[64] Buysse, D. J., Reynolds, C. F., Monk, T. H., Berman, S. R. & Kupfer, D. J. The Pittsburgh Sleep Quality Index: a new instrument for psychiatric practice and research. *Psychiatry Res.* **28**, 193–213 (1989). 2748771 10.1016/0165-1781(89)90047-4

[65] Buysse, D. J. et al. Development and validation of patient-reported outcome measures for sleep disturbance and sleep-related impairments. *Sleep* **33**, 781–792 (2010). 20550019 10.1093/sleep/33.6.781 PMC2880437

[66] Zauner, J., Hartmeyer, S. & Spitschan, M. LightLogR: reproducible analysis of personal light exposure data. *J. Open Source Softw.* **10**, 7601 (2025). 40123959 10.21105/joss.07601 PMC7617517

