# Supplementary material for: Light exposure and sleep architecture in real-world settings
Source: NPJ Biol Timing Sleep. 2026 Jul 10;3:30. doi: 10.1038/s44323-026-00087-z (PMC13354558; doi:10.1038/s44323-026-00087-z)
Supplement: Supplementary file 1 — Supplementary materials-20260316 [file 44323_2026_87_MOESM1_ESM.pdf]

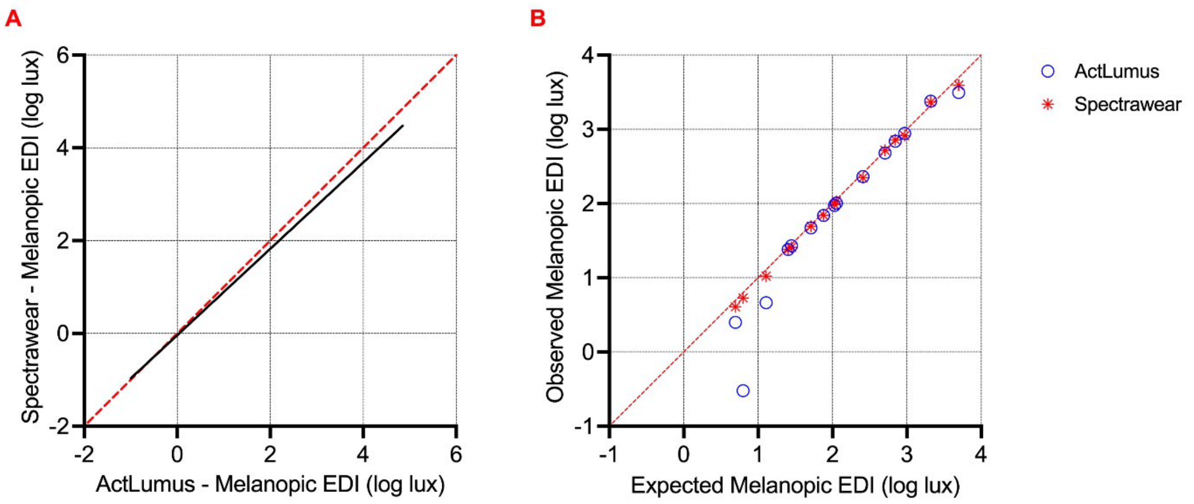

FIGURE S1: Supplementary analysis assessing correlations between light-sensing devices. An ActLumus Condor light sensor and Spectrawear were worn side-by-side on the same arm by one participant for 4.5 consecutive days (1-minute sampling interval; N = 6,730). **A)** Melanopic equivalent daylight illuminance (EDI; lux) values from both devices were compared using linear regression. The slope was 0.93, 95% CI [0.93, 0.94], $R^2 = .96$, $p < .001$. The red line indicates the equality line (slope = 1). **B)** A white LED light source was tested across seven intensity levels (0–4000 melanopic EDI lux), and fluorescent bulbs were tested across eight intensity levels (0–2500 melanopic EDI lux). Spectral irradiance was measured using a calibrated SpectroCAL MKII Spectroradiometer (Cambridge Research Systems, UK), and melanopic EDI lux values from both devices were compared using linear regression. Slopes did not differ significantly, $F(1, 26) = 3.93$, $p = .058$, and intercepts did not differ significantly, $F(1, 27) = 2.43$, $p = .131$. The pooled slope equals 1.11. The red line indicates the equality line (slope = 1).

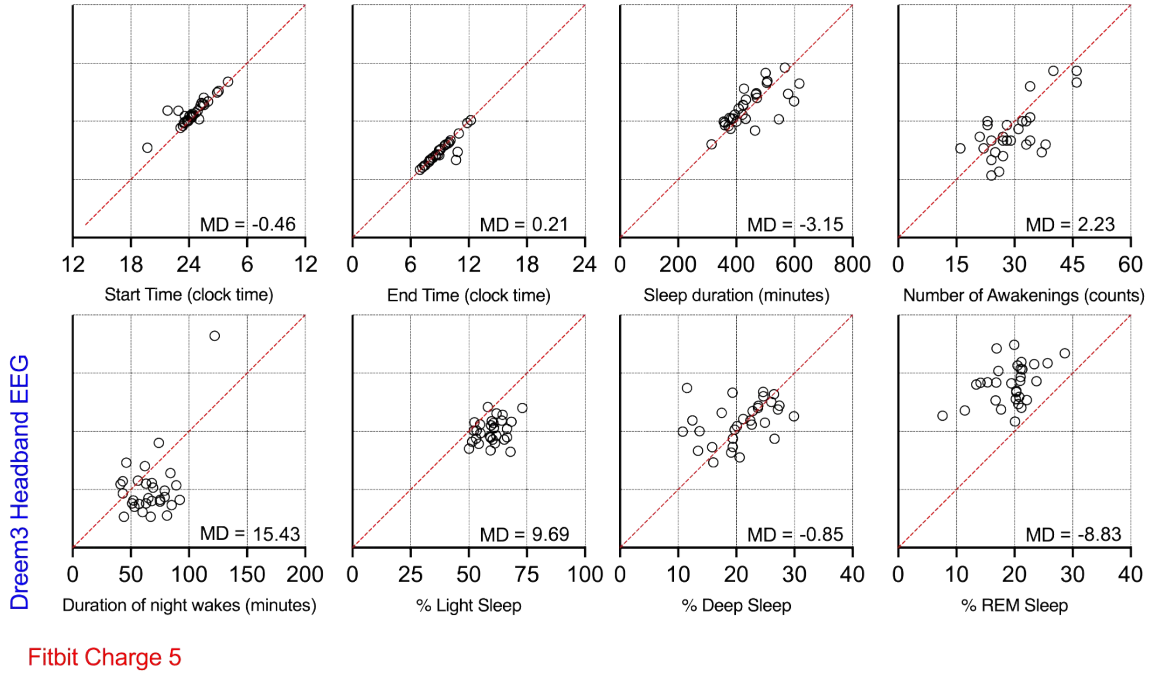

FIGURE S2: To evaluate the correspondence between sleep metrics, a Fitbit Charge 5 and a Dreem 3 EEG headband (Beacon Biosignals, Boston, MA) were worn simultaneously by two participants across 30 nights. Sleep parameters derived from both devices were compared using linear regression models. The red line indicates the equality line (slope = 1). The mean difference (MD) between devices (Fitbit – Dreem) is reported in the corner of each graph.

Supplementary Table 1. Summary of the daily and weekly light exposure variables.

	Variable	Count	Min or earliest	Max or latest	Median	Mean	SD
Daily Light Exposure	Duration above 250 lux melanopic EDI	542	0.00	685.50	186.00	207.55	141.23
	Time of last exposure >1000 lux melanopic EDI (hh:mm)	510	8:01	21:28	18:15	17:30	2.60
	Time of last exposure >1 lux melanopic EDI (hh:mm)	542	16:19	04:00	23:52	00:08	1.78
	Mean melanopic EDI 3 hours before sleep	454	0.10	104.23	2.64	6.55	12.11
	Mean melanopic EDI during the sleep period	454	0.10	8.23	0.10	0.19	0.63
Weekly Light Exposure	Duration above 250 lux melanopic EDI	89	31.00	473.00	199.17	206.09	100.19
	Mean log melanopic EDI during the 10 brightest hours	89	0.89	2.80	1.96	1.93	0.39
	Mean log melanopic EDI during the 5 dimmest hours	89	-1.00	0.35	-0.97	-0.86	0.25
	Interdaily stability	89	0.34	0.96	0.80	0.78	0.10
	Intradaily variability	89	0.18	0.60	0.31	0.31	0.08
	Mean time of last exposure >1000 lux melanopic EDI (hh:mm)	89	11:47	20:28	17:56	17:28	2.05
	Average time of last exposure >1 lux melanopic EDI (hh:mm)	89	21:28	03:35	23:59	00:08	1.32

Supplementary Table 2. Associations between light exposure and covariates.

**Response**	**Age (Over 30 - Below 30)**	**Sex (Male - Female)**	**Day Type (Weekday - Weekend)**	**MSFsc**	**Photoperiod**
time1000last	Est: 0.30	Est: 0.11	Est: -0.55	Est: 0.15	Est: 0.65
	p(adj) = 0.42	p(adj) = 0.82	p(adj) = **0.025**	p(adj) = 0.32	p(adj) = **6.92e-22**
time1last	Est: 0.12	Est: 0.27	Est: -0.50	Est: 0.41	Est: 0.14
	p(adj) = 0.80	p(adj) = 0.39	p(adj) = **8.82e-04**	p(adj) = **2.57e-05**	p(adj) = **1.57e-03**
TAT250	Est: 2.74	Est: 36.56	Est: -13.87	Est: -14.57	Est: 14.95
	p(adj) = 0.90	p(adj) = 0.20	p(adj) = 0.41	p(adj) = 0.20	p(adj) = **9.39e-04**
presleep3hr	Est: 0.42	Est: -0.35	Est: 0.57	Est: -0.11	Est: 0.53
	p(adj) = 0.87	p(adj) = 0.87	p(adj) = 0.80	p(adj) = 0.87	p(adj) = 0.15
sleepPeriodave	Est: 0.14	Est: 0.058	Est: -0.069	Est: 0.074	Est: 0.018
	p(adj) = 0.33	p(adj) = 0.76	p(adj) = 0.41	p(adj) = 0.15	p(adj) = 0.42
M10	Est: -0.068	Est: 0.10	–	Est: -0.074	Est: 0.076
	p(adj) = 0.48	p(adj) = 0.28		p(adj) = **0.030**	p(adj) = **5.69e-06**
L5	Est: 0.058	Est: -0.017	–	Est: 0.093	Est: 0.029
	p(adj) = 0.34	p(adj) = 0.71		p(adj) = **1.28e-05**	p(adj) = **2.61e-03**
IS	Est: -0.022	Est: 0.017	–	Est: -0.038	Est: 2.43e-03
	p(adj) = 0.43	p(adj) = 0.49		p(adj) = **8.48e-05**	p(adj) = 0.56
IV	Est: 0.030	Est: -0.049	–	Est: 0.026	Est: -6.41e-03
	p(adj) = 0.19	p(adj) = **0.018**		p(adj) = **9.64e-04**	p(adj) = 0.090

M10, L5, IS, and IV were compared against covariates using multivariate linear regression and the other variables were compared against covariates using linear mixed models with random participant intercepts. P-values were adjusted using the Benjamini–Hochberg procedure to control the false discovery rate (FDR). Variables: TAT250, Duration above 250 lux melanopic EDI (min); time1000last, Time of last exposure >1000 lux melanopic EDI (decimal hour); time1last, Time of last exposure >1 lux melanopic EDI (decimal hour); M10, Mean light exposure during the 10 brightest consecutive hours (log lux melanopic EDI); L5, Mean light exposure during the 5 dimmest consecutive hours (log lux melanopic EDI); IS, Interdaily stability; IV, Intradaily variability; presleep3hr, Light exposure 3 hours before sleep (lux melanopic EDI); sleepPeriodave, Average light exposure during the sleep period (lux melanopic EDI).

Supplementary Table 3a. Summary of the objective sleep variables.

Variable	Count	Min or earliest	Max or latest	Median	Mean	SD
Sleep duration (min)	547	244.00	671.00	416.00	414.29	63.69
Sleep efficiency (%)	547	56.63	96.08	87.93	87.74	3.37
Sleep time (hh:mm)	547	19:39	3:56	00:01	00:13	1.38
Wake time (hh:mm)	547	04:34	12:41	07:55	08:05	1.39
Wake count	529	11.00	62.00	29.00	29.30	8.03
Wake duration (min)	529	19.00	125.00	57.00	58.22	16.97
Deep sleep (%)	529	3.58	39.03	19.48	19.32	5.42
Rem sleep (%)	529	4.79	42.34	21.51	21.14	6.01
Light sleep (%)	529	31.95	89.01	58.92	59.54	8.52
Average deep sleep block duration (min)	529	6.00	60.50	20.67	21.93	8.78
Difference in the proportion of deep sleep between the first and last third of the sleep period	529	-0.45	0.84	0.29	0.27	0.20
Rem sleep latency (min)	529	8.50	379.50	114.50	129.10	66.13
Sleep Regularity Index	547	63.58	98.96	87.38	86.58	7.52

Supplementary Table 3b. Summary of the subjective sleep variables**.**

Variable	Count	Min or earliest	Max or latest	Median	Mean	SD
Sleep duration (min)	528	240.00	660.00	450.00	436.78	65.43
Sleep efficiency (%)	528	44.44	100.00	93.33	91.11	8.99
Sleep time (hh:mm)	528	19:05	03:30	00:00	00:11	1.35
Wake time (hh:mm)	528	04:45	12:35	07:40	07:54	1.34

Supplementary Table 4. Associations between objective sleep outcomes and covariates.

**Response**	**Age (Over 30 - Below 30)**	**Sex (Male - Female)**	**Day Type (Weekday - Weekend)**	**MSFsc**	**Photoperiod**
wakeTime	Estimate: 0.048	Estimate: -0.16	Estimate: -0.85	Estimate: 0.56	Estimate: 0.045
	p (adj) = 0.96	p (adj) = 0.64	p (adj) = **6.76e-17**	p (adj) = **3.59e-11**	p (adj) = 0.44
sleepTime	Estimate: 0.096	Estimate: 0.25	Estimate: -0.55	Estimate: 0.53	Estimate: 0.082
	p (adj) = 0.85	p (adj) = 0.44	p (adj) = **1.01e-06**	p (adj) = **5.16e-11**	p (adj) = 0.064
sleepEfficiency	Estimate: -0.35	Estimate: -1.75	Estimate: -0.079	Estimate: -0.29	Estimate: 0.099
	p (adj) = 0.82	p (adj) = **2.71e-03**	p (adj) = 0.96	p (adj) = 0.36	p (adj) = 0.57
sleepDuration	Estimate: -4.30	Estimate: -29.34	Estimate: -15.88	Estimate: -0.17	Estimate: -1.383
	p (adj) = 0.86	p (adj) = **7.82e-03**	p (adj) = 0.063	p (adj) = 0.99	p (adj) = 0.67
deepPerc	Estimate: -0.93	Estimate: 0.38	Estimate: 0.51	Estimate: 0.084	Estimate: -0.33
	p (adj) = 0.58	p (adj) = 0.85	p (adj) = 0.61	p (adj) = 0.96	p (adj) = 0.13
deepRatiodiff	Estimate: -5.13e-03	Estimate: -0.056	Estimate: -0.033	Estimate: -1.14e-03	Estimate: -1.08e-03
	p (adj) = 0.96	p (adj) = 0.19	p (adj) = 0.36	p (adj) = 0.99	p (adj) = 0.96
wakeCount	Estimate: 0.82	Estimate: -0.23	Estimate: -1.38	Estimate: 0.53	Estimate: 0.065
	p (adj) = 0.85	p (adj) = 0.96	p (adj) = 0.25	p (adj) = 0.59	p (adj) = 0.96
remPerc	Estimate: -0.47	Estimate: -1.67	Estimate: -0.041	Estimate: -0.33	Estimate: -0.027
	p (adj) = 0.85	p (adj) = 0.25	p (adj) = 0.99	p (adj) = 0.61	p (adj) = 0.96
remLatency	Estimate: -0.66	Estimate: -15.26	Estimate: -8.45	Estimate: -0.048	Estimate: 0.018
	p (adj) = 0.99	p (adj) = 0.27	p (adj) = 0.54	p (adj) = 0.99	p (adj) = 0.99
deepCons	Estimate: -0.45	Estimate: 0.77	Estimate: -0.61	Estimate: 0.21	Estimate: -0.31
	p (adj) = 0.86	p (adj) = 0.69	p (adj) = 0.83	p (adj) = 0.85	p (adj) = 0.27
lightPerc	Estimate: 1.40	Estimate: 1.29	Estimate: -0.47	Estimate: 0.24	Estimate: 0.35
	p (adj) = 0.59	p (adj) = 0.59	p (adj) = 0.85	p (adj) = 0.85	p (adj) = 0.40
wakeMinute	Estimate: -0.58	Estimate: 3.55	Estimate: -1.98	Estimate: 1.13	Estimate: -0.65
	p (adj) = 0.96	p (adj) = 0.44	p (adj) = 0.53	p (adj) = 0.57	p (adj) = 0.44
SRI	Estimate: 3.28	Estimate: -0.23	–	Estimate: -0.30	Estimate: -0.12
	p (adj) = 0.32	p (adj) = 0.90		p (adj) = 0.90	p (adj) = 0.90

All outcomes except SRI were compared against covariates using multivariate linear mix model, SRI was compared against covariates using multivariate linear regression. P-values were adjusted using the Benjamini–Hochberg procedure to control the false discovery rate (FDR). Variables: sleepDuration, Sleep duration (min); sleepEfficiency, Sleep efficiency (%); sleepTime, Sleep onset time (decimal hour); wakeTime, Wake time (decimal hour); wakeCount, Wake count; wakeMinute, Wake duration (min); deepPerc, Deep sleep (%); remPerc, REM sleep (%); lightPerc, Light sleep (%); deepCons, Average deep sleep block duration (min); deepRatiodiff, Difference in the deep sleep ratio between first and last 1/3 of sleep duration; remLatency, REM sleep latency (min); SRI, sleep regularity index (%).

Supplementary Table 5. Associations between daily light exposure and objective sleep variables assessed using linear mixed models.

Response	Predictor	Estimate	SE	t	Std β	η²	p	p (adj)
sleepTime	time1last	0.49	0.029	16.78	0.56	0.38	**1.71e-49**	**1.03e-47**
sleepDuration	time1last	-14.011	2.00	-7.023	-0.35	0.099	**8.07e-12**	**2.42e-10**
wakeTime	time1last	0.22	3.20e-02	6.64	0.25	0.089	**8.81e-11**	**1.76e-09**
sleepTime	presleep3hr	-0.023	3.88e-03	-5.94	-0.20	0.077	**5.97e-09**	**8.96e-08**
wakeCount	time1last	-1.44	0.25	-5.80	-0.29	0.072	**1.26e-08**	**1.51e-07**
sleepDuration	presleep3hr	1.01	0.23	4.39	0.19	0.042	**1.42e-05**	**1.42e-04**
wakeMinute	time1last	-1.74	0.55	-3.17	-0.17	0.022	**1.63e-03**	**0.014**
wakeMinute	presleep3hr	0.17	0.060	2.79	0.12	0.019	**5.52e-03**	**0.041**
sleepTime	TAT250	-1.03e-03	3.89e-04	-2.65	-0.11	1.50e-02	**8.43e-03**	0.056
wakeTime	sleepPeriodave	-1.22e-01	7.60e-02	-1.61	-0.057	5.75e-03	0.11	0.59
remLatency	presleep3hr	4.10e-01	2.56e-01	1.60	0.076	5.85e-03	0.11	0.59
deepPerc	TAT250	-2.89e-03	1.94e-03	-1.49	-0.075	5.11e-03	0.14	0.59
lightPerc	TAT250	4.59e-03	3.10e-03	1.48	0.075	5.05e-03	0.14	0.59
wakeTime	TAT250	-5.26e-04	3.62e-04	-1.46	-0.055	4.66e-03	0.15	0.59
deepCons	time1last	0.42	0.29	1.46	0.079	5.99e-03	0.15	0.59
wakeCount	presleep3hr	0.039	0.028	1.41	0.061	4.89e-03	0.16	0.60
remLatency	TAT250	0.030	0.024	1.22	0.063	3.77e-03	0.22	0.79
wakeMinute	time1000last	-0.46	0.39	-1.17	-0.071	3.39e-03	0.24	0.80
wakeCount	TAT250	-3.19e-03	2.79e-03	-1.14	-0.056	2.99e-03	0.25	0.80
remLatency	time1last	-2.36	2.27	-1.041	-0.058	2.68e-03	0.30	0.85
sleepDuration	TAT250	0.022	0.022	0.99	0.049	2.22e-03	0.32	0.85
deepRatiodiff	time1000last	4.71e-03	4.81e-03	0.98	0.062	2.33e-03	0.33	0.85
sleepDuration	sleepPeriodave	-4.46e+00	4.71e+00	-0.95	-0.044	1.99e-03	0.34	0.85
wakeTime	presleep3hr	-3.38e-03	3.69e-03	-0.92	-0.030	2.02e-03	0.36	0.85
deepCons	presleep3hr	0.030	0.034	0.90	0.043	1.84e-03	0.37	0.85
remPerc	time1last	-0.18	0.20	-0.88	-0.048	1.79e-03	0.38	0.85
deepPerc	time1000last	-0.10	0.13	-0.79	-0.049	1.53e-03	0.43	0.85
remPerc	TAT250	-1.71e-03	2.19e-03	-0.78	-0.040	1.40e-03	0.44	0.85
remPerc	sleepPeriodave	-0.35	0.45	-0.77	-0.037	1.36e-03	0.44	0.85
remLatency	time1000last	-1.16	1.63	-0.71	-0.046	1.25e-03	0.48	0.85
lightPerc	time1000last	0.15	0.20	0.71	0.045	1.23e-03	0.48	0.85
deepPerc	presleep3hr	-1.30e-02	2.00e-02	-0.68	-0.031	1.09e-03	0.50	0.85
deepPerc	sleepPeriodave	2.63e-01	3.97e-01	0.66	0.032	1.01e-03	0.51	0.85
wakeMinute	TAT250	3.95e-03	5.99e-03	0.66	0.033	9.97e-04	0.51	0.85
remLatency	sleepPeriodave	3.18	5.029	0.63	0.031	9.57e-04	0.53	0.85
lightPerc	time1last	0.18	0.29	0.63	0.034	9.11e-04	0.53	0.85
wakeCount	time1000last	-0.11	0.18	-0.62	-0.037	9.71e-04	0.54	0.85
deepRatiodiff	presleep3hr	4.63e-04	7.55e-04	0.61	0.029	8.70e-04	0.54	0.85
lightPerc	presleep3hr	0.018	0.032	0.57	0.026	7.69e-04	0.57	0.88
deepCons	TAT250	1.64e-03	3.11e-03	0.53	0.027	8.21e-04	0.60	0.88
sleepEfficiency	time1000last	4.20e-02	0.080	0.53	0.031	6.46e-04	0.60	0.88
deepCons	sleepPeriodave	-3.26e-01	6.49e-01	-0.50	-0.024	6.39e-04	0.62	0.88
deepRatiodiff	sleepPeriodave	-7.09e-03	0.015	-0.47	-0.023	5.24e-04	0.64	0.89

sleepEfficiency	sleepPeriodave	-0.11	0.25	-0.45	-0.020	4.47e-04	0.65	0.89
deepRatiodiff	time1last	2.57e-03	6.76e-03	0.38	0.021	3.43e-04	0.70	0.90
sleepTime	sleepPeriodave	-0.031	0.083	-0.37	-0.014	3.08e-04	0.71	0.90
deepCons	time1000last	-0.078	0.21	-0.37	-0.023	3.47e-04	0.72	0.90
sleepEfficiency	time1last	-3.90e-02	1.11e-01	-0.36	-0.018	2.79e-04	0.72	0.90
remPerc	time1000last	-0.045	0.15	-0.31	-0.019	2.36e-04	0.76	0.93
wakeMinute	sleepPeriodave	0.34	1.22	0.28	0.013	1.79e-04	0.78	0.94
sleepTime	time1000last	5.97e-03	0.026	0.23	0.011	1.29e-04	0.82	0.95
sleepEfficiency	TAT250	-2.63e-04	1.18e-03	-0.22	-0.011	1.13e-04	0.82	0.95
remPerc	presleep3hr	-4.62e-03	0.022	-0.21	-9.38e-03	1.02e-04	0.84	0.95
sleepEfficiency	presleep3hr	-1.70e-03	1.20e-02	-0.14	-5.98e-03	4.33e-05	0.89	0.95
wakeCount	sleepPeriodave	-7.70e-02	0.57	-0.14	-6.25e-03	4.18e-05	0.89	0.95
lightPerc	sleepPeriodave	8.40e-02	6.36e-01	0.13	6.33e-03	4.00e-05	0.90	0.95
deepRatiodiff	TAT250	9.25e-06	7.29e-05	0.13	6.55e-03	3.88e-05	0.90	0.95
wakeTime	time1000last	-1.85e-03	2.40e-02	-0.077	-3.49e-03	1.45e-05	0.94	0.97
sleepDuration	time1000last	-3.40e-02	1.49	-0.023	-1.36e-03	1.20e-06	0.98	1.00
deepPerc	time1last	-7.27e-04	0.18	-4.04e-03	-2.18e-04	3.76e-08	1.00	1.00

Variables: sleepDuration, Sleep duration (min); sleepEfficiency, Sleep efficiency (%); sleepTime, Sleep onset time (decimal hour); wakeTime, Wake time (decimal hour); wakeCount, Number of awakenings during sleep; wakeMinute, Wake duration during sleep (min); deepPerc, Deep sleep (%); remPerc, REM sleep (%); lightPerc, Light sleep (%); deepCons, Average deep sleep block duration (min); deepRatiodiff, Difference in the deep sleep ratio between first and last 1/3 of sleep duration; remLatency, REM sleep latency (min); TAT250, Duration above 250 lux melanopic EDI (min); time1000last, Time of last exposure >1000 lux melanopic EDI (decimal hour); time1last, Time of last exposure >1 lux melanopic EDI (decimal hour); presleep3hr, Mean light exposure 3 hours before sleep (lux melanopic EDI); sleepPeriodave, Mean light exposure during the sleep period (lux melanopic EDI)

Supplementary Table 6. Associations between weekly light exposure and objective sleep variables assessed using linear regression models.

Response	Predictor	Estimate	SE	t	Std β	Adj. R²	p	p (adj)
sleepTime	time1lastave	0.45	0.070	6.37	0.52	0.67	**9.79e-09**	**8.91e-07**
wakeTime	time1lastave	0.34	0.077	4.38	0.41	0.56	**3.44e-05**	**1.56e-03**
sleepTime	IV	4.33	1.03	4.20	0.32	0.59	**6.66e-05**	**2.02e-03**
sleepDuration	IV	-191.15	51.20	-3.73	-0.40	0.20	**3.46e-04**	**7.86e-03**
deepRatiodiff	IV	-0.52	0.16	-3.23	-0.35	0.17	**1.80e-03**	**0.026**
wakeTime	TAT250	-2.87e-03	9.02e-04	-3.19	-0.26	0.52	**2.03e-03**	**0.026**
deepRatiodiff	IS	0.42	0.13	3.19	0.35	0.17	**2.03e-03**	**0.026**
sleepTime	IS	-2.72	0.88	-3.088	-0.25	0.55	**2.74e-03**	**0.029**
sleepTime	M10	-0.76	0.25	-3.068	-0.26	0.55	**2.91e-03**	**0.029**
deepRatiodiff	M10	0.11	0.039	2.81	0.33	0.15	**6.17e-03**	0.056
sleepTime	TAT250	-2.48e-03	9.12e-04	-2.72	-0.22	0.54	**7.96e-03**	0.066
sleepTime	L5	1.041	0.41	2.54	0.23	0.54	**0.013**	0.098
remPerc	IS	10.72	4.47	2.40	0.28	0.072	**0.019**	0.13
sleepEfficiency	TAT250	5.72e-03	2.42e-03	2.37	0.25	0.24	**0.020**	0.13
wakeTime	M10	-0.59	0.25	-2.34	-0.21	0.50	**0.022**	0.13
wakeCount	IV	-18.48	8.55	-2.16	-0.26	5.00e-03	**0.034**	0.19
sleepEfficiency	M10	1.42	0.66	2.15	0.24	0.23	**0.035**	0.19
SRI	TAT250	-0.019	9.06e-03	-2.06	-0.24	0.039	**0.042**	0.21
remPerc	L5	-3.98	2.04	-1.95	-0.25	0.050	0.055	0.26
deepPerc	time1lastave	0.66	0.35	1.87	0.26	0.063	0.065	0.30
SRI	time1000lastave	-1.23	0.68	-1.82	-0.32	0.028	0.072	0.31
wakeTime	IS	-1.60	0.92	-1.75	-0.15	0.48	0.085	0.35
wakeCount	IS	12.20	7.10	1.72	0.21	-0.016	0.089	0.35
sleepDuration	time1lastave	-6.64	4.09	-1.62	-0.22	0.089	0.11	0.41
wakeTime	L5	0.67	0.42	1.60	0.15	0.48	0.11	0.41
lightPerc	IS	-10.14	6.46	-1.57	-0.19	0.031	0.12	0.41
deepRatiodiff	time1000lastave	0.016	0.010	1.57	0.27	0.095	0.12	0.41
sleepDuration	IS	69.11	44.66	1.55	0.18	0.086	0.13	0.41
wakeMinute	TAT250	-0.020	0.013	-1.52	-0.18	0.059	0.13	0.42
SRI	M10	-3.68	2.50	-1.47	-0.19	0.015	0.15	0.44
sleepTime	time1000lastave	-0.093	0.070	-1.34	-0.17	0.51	0.19	0.55
deepCons	L5	-2.87	2.21	-1.30	-0.17	0.024	0.20	0.56
sleepDuration	time1000lastave	4.38	3.41	1.28	0.22	0.078	0.20	0.56
SRI	L5	5.21	4.11	1.27	0.17	8.37e-03	0.21	0.56
deepRatiodiff	TAT250	1.82e-04	1.46e-04	1.25	0.14	0.085	0.22	0.56
sleepDuration	M10	15.33	12.55	1.22	0.15	0.077	0.23	0.57
wakeCount	time1000lastave	0.63	0.54	1.18	0.22	-0.035	0.24	0.59
deepCons	IV	-6.96	5.96	-1.17	-0.14	0.020	0.25	0.59
wakeCount	M10	2.40	2.09	1.14	0.15	-0.036	0.26	0.59
remLatency	L5	-21.60	18.98	-1.14	-0.15	-8.65e-03	0.26	0.59
sleepDuration	L5	-22.63	20.57	-1.10	-0.14	0.074	0.27	0.61
deepPerc	L5	1.83	1.78	1.03	0.13	0.035	0.31	0.67
wakeCount	time1lastave	-0.65	0.65	-1.00	-0.15	-0.040	0.32	0.68
remPerc	IV	-5.50	5.60	-0.98	-0.12	0.017	0.33	0.68
lightPerc	IV	7.48	7.93	0.94	0.11	0.012	0.35	0.70
remLatency	IV	-47.97	51.32	-0.94	-0.11	-0.014	0.35	0.70
remPerc	time1lastave	-0.38	0.42	-0.91	-0.13	0.015	0.36	0.70
deepPerc	time1000lastave	-0.27	0.30	-0.90	-0.16	0.032	0.37	0.70
sleepEfficiency	IV	-2.64	2.96	-0.89	-0.096	0.19	0.38	0.70

sleepEfficiency	IS	2.13	2.43	0.88	0.094	0.19	0.38	0.70
deepCons	IS	4.13	4.92	0.84	0.10	0.012	0.40	0.70
wakeTime	IV	0.95	1.14	0.84	0.073	0.47	0.41	0.70
wakeMinute	M10	-3.12	3.77	-0.83	-0.10	0.041	0.41	0.70
remLatency	time1lastave	-2.95	3.84	-0.77	-0.11	-0.017	0.44	0.72
lightPerc	TAT250	5.10e-03	6.82e-03	0.75	0.090	8.02e-03	0.46	0.72
deepRatiodiff	L5	-0.047	0.063	-0.75	-0.096	0.073	0.46	0.72
SRI	IS	6.62	9.04	0.73	0.088	-4.36e-03	0.47	0.72
lightPerc	L5	2.15	2.95	0.73	0.097	7.67e-03	0.47	0.72
wakeMinute	IV	-11.36	15.71	-0.72	-0.086	0.039	0.47	0.72
deepRatiodiff	time1lastave	9.10e-03	0.013	0.71	0.098	0.073	0.48	0.72
remLatency	TAT250	0.030	0.044	0.68	0.083	-0.019	0.50	0.74
deepCons	time1000lastave	-0.24	0.37	-0.66	-0.12	9.09e-03	0.51	0.74
sleepEfficiency	time1lastave	-0.15	0.22	-0.66	-0.083	0.19	0.51	0.74
sleepEfficiency	time1000lastave	0.12	0.19	0.62	0.10	0.19	0.54	0.76
remPerc	TAT250	-2.80e-03	4.83e-03	-0.58	-0.069	9.37e-03	0.56	0.78
lightPerc	time1000lastave	0.28	0.49	0.57	0.10	5.09e-03	0.57	0.78
deepPerc	TAT250	-2.31e-03	4.13e-03	-0.56	-0.066	0.026	0.58	0.78
wakeMinute	IS	-7.07	12.92	-0.55	-0.066	0.036	0.59	0.78
SRI	IV	5.77	11.06	0.52	0.062	-7.53e-03	0.60	0.80
wakeCount	L5	-1.59	3.26	-0.49	-0.067	-0.050	0.63	0.81
remLatency	M10	5.90	12.36	0.48	0.062	-0.022	0.63	0.81
lightPerc	time1lastave	-0.28	0.60	-0.47	-0.066	3.77e-03	0.64	0.81
deepCons	M10	0.63	1.44	0.44	0.056	6.15e-03	0.66	0.81
SRI	time1lastave	0.36	0.83	0.43	0.060	-8.59e-03	0.67	0.81
sleepEfficiency	L5	-0.48	1.11	-0.43	-0.051	0.19	0.67	0.81
deepPerc	IV	-1.98	4.82	-0.41	-0.049	0.024	0.68	0.82
deepCons	time1lastave	0.18	0.45	0.39	0.056	5.66e-03	0.70	0.82
wakeMinute	time1lastave	0.40	1.18	0.34	0.048	0.034	0.73	0.86
wakeTime	time1000lastave	-0.020	0.071	-0.29	-0.038	0.46	0.77	0.87
wakeMinute	time1000lastave	-0.27	0.97	-0.28	-0.050	0.033	0.78	0.87
remLatency	time1000lastave	-0.88	3.18	-0.28	-0.051	-0.024	0.78	0.87
remPerc	M10	-0.37	1.35	-0.28	-0.035	6.17e-03	0.78	0.87
deepCons	TAT250	-1.37e-03	5.16e-03	-0.27	-0.032	4.63e-03	0.79	0.87
wakeCount	TAT250	-1.65e-03	7.55e-03	-0.22	-0.027	-0.053	0.83	0.90
deepPerc	M10	0.23	1.16	0.20	0.025	0.022	0.85	0.91
deepPerc	IS	-0.58	3.96	-0.15	-0.018	0.022	0.88	0.94
sleepDuration	TAT250	6.18e-03	0.046	0.13	0.015	0.060	0.89	0.94
wakeMinute	L5	0.64	5.85	0.11	0.014	0.032	0.91	0.94
remLatency	IS	-4.45	42.39	-0.11	-0.013	-0.025	0.92	0.94
lightPerc	M10	0.15	1.91	0.077	9.90e-03	1.15e-03	0.94	0.95
remPerc	time1000lastave	-0.011	0.35	-0.033	-5.97e-03	5.23e-03	0.97	0.97

Variables: sleepDuration, Sleep duration (min); sleepEfficiency, Sleep efficiency (%); sleepTime, Sleep onset time (decimal hour); wakeTime, Wake time (decimal hour); wakeCount, Number of awakenings during sleep; wakeMinute, Wake duration during sleep (min); deepPerc, Deep sleep (%); remPerc, REM sleep (%); lightPerc, Light sleep (%); deepCons, Average deep sleep block duration (min); deepRatiodiff, Difference in the deep sleep ratio between first and last 1/3 of sleep duration; remLatency, REM sleep latency (min); TAT250, Duration above 250 lux melanopic EDI (min); M10, Mean light exposure during the 10 brightest consecutive hours (log lux melanopic EDI); L5, Mean light exposure during the 5 dimmest consecutive hours (log lux melanopic EDI); IS, Interdaily stability; IV, Intradaily variability; time1000lastave, Average time of last exposure >1000 lux melanopic EDI (decimal hour); time1lastave, Average time of last exposure >1 lux melanopic EDI (decimal hour); SRI, sleep regularity index (%).

Supplementary Table 7. Associations between objective sleep and subjective sleep variables assessed using linear mixed models.

Response	Predictor	Estimate	SE	t	Std β	η²	p	p (adj)
wakeTime_fitbit	wakeTime_diary	0.95	0.019	48.79	0.93	0.83	**1.08e-189**	**5.16e-188**
sleepTime_fitbit	sleepTime_diary	0.84	0.026	32.96	0.85	0.75	**7.82e-110**	**1.88e-108**
sleepDuration_fitbit	sleepDuration_diary	0.65	0.033	19.99	0.67	0.43	**1.35e-66**	**2.16e-65**
sleepTime_fitbit	wakeTime_diary	0.52	0.039	13.45	0.52	0.28	**4.12e-35**	**4.94e-34**
wakeCount	sleepDuration_diary	0.056	4.44e-03	12.58	0.46	0.24	**1.08e-31**	**1.04e-30**
wakeTime_fitbit	sleepTime_diary	0.50	0.040	12.64	0.50	0.27	**2.55e-31**	**2.04e-30**
wakeTime_fitbit	sleepDuration_diary	6.99e-03	7.18e-04	9.74	0.33	0.16	**1.25e-20**	**8.54e-20**
sleepDuration_fitbit	wakeTime_diary	21.49	2.24	9.62	0.46	0.17	**4.68e-20**	**2.81e-19**
wakeCount	wakeTime_diary	2.12	0.28	7.60	0.36	0.11	**1.61e-13**	**8.60e-13**
sleepDuration_fitbit	sleepTime_diary	-16.81	2.27	-7.41	-0.36	0.12	**7.44e-13**	**3.57e-12**
sleepTime_fitbit	sleepDuration_diary	-5.13e-03	7.44e-04	-6.90	-0.25	0.086	**1.57e-11**	**6.85e-11**
sleepEfficiency_fitbit	sleepEfficiency_diary	0.10	0.016	6.28	0.27	0.070	**7.06e-10**	**2.83e-09**
wakeCount	sleepTime_diary	-1.74	0.30	-5.79	-0.29	0.065	**1.25e-08**	**4.63e-08**
wakeMinute	wakeTime_diary	3.52	0.62	5.64	0.28	0.074	**3.15e-08**	**1.08e-07**
wakeMinute	sleepDuration_diary	0.051	0.011	4.51	0.20	0.038	**8.02e-06**	**2.57e-05**
wakeMinute	sleepEfficiency_diary	-0.39	0.088	-4.41	-0.20	0.038	**1.30e-05**	**3.89e-05**
wakeMinute	sleepTime_diary	-1.68	0.67	-2.51	-0.13	0.016	**0.012**	**0.035**
sleepEfficiency_fitbit	sleepDuration_diary	5.39e-03	2.20e-03	2.45	0.10	0.011	**0.014**	**0.038**
remPerc	sleepTime_diary	-0.57	0.23	-2.44	-0.13	0.015	**0.015**	**0.038**
remPerc	sleepDuration_diary	8.58e-03	4.01e-03	2.14	0.093	8.92e-03	**0.033**	0.078
deepRatiodiff	sleepEfficiency_diary	2.08e-03	1.05e-03	1.98	0.094	8.56e-03	**0.049**	0.11
lightPerc	sleepTime_diary	0.64	0.33	1.93	0.10	9.25e-03	0.054	0.12
deepRatiodiff	sleepDuration_diary	2.61e-04	1.38e-04	1.89	0.086	7.10e-03	0.059	0.12
remLatency	sleepTime_diary	-4.54	2.45	-1.86	-0.091	0.015	0.065	0.13
remPerc	sleepEfficiency_diary	0.050	0.031	1.62	0.075	5.24e-03	0.11	0.20
remLatency	sleepEfficiency_diary	-0.49	0.35	-1.40	-0.066	5.01e-03	0.16	0.30
lightPerc	sleepEfficiency_diary	-0.057	0.044	-1.30	-0.060	3.34e-03	0.20	0.35
sleepEfficiency_fitbit	wakeTime_diary	-0.16	0.12	-1.27	-0.062	3.45e-03	0.21	0.35
wakeTime_fitbit	sleepEfficiency_diary	-7.48e-03	6.08e-03	-1.23	-0.049	2.90e-03	0.22	0.36
lightPerc	wakeTime_diary	0.37	0.32	1.17	0.059	3.12e-03	0.24	0.39
remLatency	sleepDuration_diary	0.047	0.046	1.02	0.047	2.23e-03	0.31	0.48
deepPerc	sleepDuration_diary	-3.48e-03	3.61e-03	-0.97	-0.042	1.82e-03	0.34	0.50
sleepEfficiency_fitbit	sleepTime_diary	0.12	0.13	0.95	0.047	1.95e-03	0.34	0.50
remPerc	wakeTime_diary	-0.21	0.23	-0.93	-0.047	2.01e-03	0.35	0.50
lightPerc	sleepDuration_diary	-5.14e-03	5.66e-03	-0.91	-0.040	1.61e-03	0.37	0.50
deepRatiodiff	wakeTime_diary	6.62e-03	7.49e-03	0.88	0.045	2.27e-03	0.38	0.50
deepPerc	wakeTime_diary	-0.16	0.20	-0.79	-0.040	1.47e-03	0.43	0.56
deepCons	wakeTime_diary	0.23	0.31	0.74	0.035	2.44e-03	0.46	0.58
deepRatiodiff	sleepTime_diary	-2.81e-03	7.69e-03	-0.37	-0.019	4.37e-04	0.72	0.87
deepCons	sleepDuration_diary	-2.19e-03	6.13e-03	-0.36	-0.016	2.95e-04	0.72	0.87
deepPerc	sleepTime_diary	-0.069	0.21	-0.33	-0.017	2.86e-04	0.74	0.87
deepCons	sleepEfficiency_diary	-0.012	0.046	-0.27	-0.012	2.12e-04	0.79	0.90
sleepDuration_fitbit	sleepEfficiency_diary	0.079	0.33	0.24	0.011	1.18e-04	0.81	0.90

deepPerc	sleepEfficiency_diary	6.10e-03	0.028	0.22	0.010	9.70e-05	0.83	0.90
wakeCount	sleepEfficiency_diary	8.04e-03	0.040	0.20	9.08e-03	8.04e-05	0.84	0.90
deepCons	sleepTime_diary	0.035	0.32	0.11	5.27e-03	6.11e-05	0.91	0.95
sleepTime_fitbit	sleepEfficiency_diary	5.57e-04	6.01e-03	0.093	3.72e-03	1.66e-05	0.93	0.95
remLatency	wakeTime_diary	-0.12	2.43	-0.051	-2.51e-03	9.77e-06	0.96	0.96

Variables: sleepDuration, Sleep duration (min); sleepEfficiency, Sleep efficiency (%); sleepTime, Sleep onset time (decimal hour); wakeTime, Wake time (decimal hour); wakeCount, Wake count; wakeMinute, Wake duration (min); deepPerc, Deep sleep (%); remPerc, REM sleep (%); lightPerc, Light sleep (%); deepCons, Average deep sleep block duration (min); deepRatiodiff, Difference in the deep sleep ratio between first and last 1/3 of sleep duration; remLatency, REM sleep latency (min).

Supplementary Table 8. Associations between objective sleep and sleep perception variables assessed using linear mixed models.

Response	Predictor	Estimate	SE	t	Std β	η²	p	p (adj)
sleepEfficiency	sleepEfficiency_diff	-0.13	0.024	-5.47	-0.22	0.054	**7.14e-08**	**1.71e-06**
sleepEfficiency	sleepDuration_diff	-0.018	3.45e-03	-5.19	-0.21	0.049	**3.01e-07**	**3.62e-06**
wakeMinute	sleepEfficiency_diff	0.66	0.13	5.11	0.21	0.049	**4.63e-07**	**3.70e-06**
remPerc	sleepEfficiency_diff	-0.15	0.045	-3.29	-0.14	0.021	**1.06e-03**	**6.36e-03**
wakeMinute	sleepDuration_diff	0.055	0.019	2.92	0.13	0.017	**3.65e-03**	**0.018**
lightPerc	sleepDuration_diff	0.027	9.33e-03	2.85	0.12	0.016	**4.54e-03**	**0.018**
wakeTime	sleepDuration_diff	2.95e-03	1.14e-03	2.60	0.090	0.013	**9.69e-03**	**0.03**
lightPerc	sleepEfficiency_diff	0.17	0.064	2.59	0.11	0.013	**9.90e-03**	**0.03**
remPerc	sleepDuration_diff	-0.017	6.63e-03	-2.53	-0.11	0.012	**0.012**	**0.031**
deepRatiodiff	sleepEfficiency_diff	-3.36e-03	1.58e-03	-2.13	-0.094	8.78e-03	**0.034**	0.082
deepCons	sleepDuration_diff	-0.019	0.010	-1.87	-0.086	7.20e-03	0.062	0.14
deepRatiodiff	sleepDuration_diff	-4.14e-04	2.30e-04	-1.80	-0.081	6.34e-03	0.072	0.14
deepPerc	sleepDuration_diff	-9.94e-03	5.99e-03	-1.66	-0.073	5.40e-03	0.097	0.18
sleepTime	sleepDuration_diff	1.80e-03	1.18e-03	1.53	0.053	4.44e-03	0.13	0.22
wakeTime	sleepEfficiency_diff	0.010	8.11e-03	1.25	0.041	3.07e-03	0.21	0.34
remLatency	sleepEfficiency_diff	0.54	0.54	0.99	0.045	1.94e-03	0.32	0.47
wakeCount	sleepDuration_diff	8.17e-03	8.42e-03	0.97	0.040	1.90e-03	0.33	0.47
sleepDuration	sleepEfficiency_diff	-0.35	0.49	-0.70	-0.030	9.31e-04	0.48	0.65
sleepTime	sleepEfficiency_diff	4.94e-03	8.38e-03	0.59	0.020	6.70e-04	0.56	0.65
sleepDuration	sleepDuration_diff	-0.040	0.069	-0.58	-0.025	6.38e-04	0.56	0.65
remLatency	sleepDuration_diff	-0.044	0.078	-0.57	-0.026	6.40e-04	0.57	0.65
deepPerc	sleepEfficiency_diff	-0.015	0.041	-0.38	-0.016	2.79e-04	0.71	0.77
wakeCount	sleepEfficiency_diff	0.014	0.058	0.24	9.62e-03	1.17e-04	0.81	0.85
deepCons	sleepEfficiency_diff	-7.28e-03	0.072	-0.10	-4.57e-03	2.12e-05	0.92	0.92

Variables: sleepDuration, Sleep duration (min); sleepEfficiency, Sleep efficiency (%); sleepTime, Sleep onset time (decimal hour); wakeTime, Wake time (decimal hour); wakeCount, Wake count; wakeMinute, Wake duration (min); deepPerc, Deep sleep (%); remPerc, REM sleep (%); lightPerc, Light sleep (%); deepCons, Average deep sleep block duration (min); deepRatiodiff, Difference in the deep sleep ratio between first and last 1/3 of sleep duration; remLatency, REM sleep latency (min); sleepDuration_diff, absolute of (Sleep diary sleep duration – Fitbit sleep duration); sleepEfficiency_diff, absolute of (Sleep diary sleep efficiency – Fitbit sleep efficiency).

Supplementary Table 9a. Associations between daily light and sleep perception variables assessed using linear mixed models.

Response	Predictor	Estimate	SE	t	Std β	η^2	p	p (adj)
sleepEfficiency_diff	presleep3hr	0.048	0.021	2.29	0.11	0.012	**0.022**	0.22
sleepDuration_diff	sleepPeriodave	4.11	3.56	1.15	0.054	3.05e-03	0.25	0.71
sleepDuration_diff	time1last	-1.31	1.31	-1.00	-0.053	2.39e-03	0.32	0.71
sleepEfficiency_diff	TAT250	1.96e-03	2.01e-03	0.98	0.050	2.28e-03	0.33	0.71
sleepEfficiency_diff	sleepPeriodave	-0.48	0.52	-0.92	-0.044	1.94e-03	0.36	0.71
sleepEfficiency_diff	time1000last	-0.11	0.14	-0.78	-0.049	1.44e-03	0.44	0.73
sleepDuration_diff	presleep3hr	-0.081	0.15	-0.55	-0.025	6.94e-04	0.58	0.83
sleepDuration_diff	time1000last	0.31	0.93	0.33	0.020	2.64e-04	0.74	0.93
sleepEfficiency_diff	time1last	0.014	0.19	0.073	3.92e-03	1.26e-05	0.94	0.96
sleepDuration_diff	TAT250	-7.09e-04	0.014	-0.051	-2.59e-03	6.50e-06	0.96	0.96

Variables: sleepDuration_diff, absolute of (Sleep diary sleep duration – Fitbit sleep duration); sleepEfficiency_diff, absolute of (Sleep diary sleep efficiency – Fitbit sleep efficiency); TAT250, Duration above 250 lux melanopic EDI (min); time1000last, Time of last exposure >1000 lux melanopic EDI (decimal hour); time1last, Time of last exposure >1 lux melanopic EDI (decimal hour); presleep3hr, Light exposure 3 hours before sleep (lux melanopic EDI); sleepPeriodave, Average light exposure during the sleep period (lux melanopic EDI).

Supplementary Table 9b. Associations between weekly light exposure and sleep perception variables assessed using linear regression models.

Response	Predictor	Estimate	SE	t	Std β	Adj. R^2	p	p (adj)
sleepDuration_diff	M10	-15.00	8.27	-1.81	-0.22	0.14	0.073	0.72
sleepEfficiency_diff	TAT250	-5.96e-03	3.84e-03	-1.55	-0.19	0.031	0.13	0.72
sleepDuration_diff	TAT250	-0.042	0.029	-1.44	-0.16	0.13	0.16	0.72
sleepEfficiency_diff	IS	3.34	3.74	0.89	0.11	0.012	0.38	0.91
sleepDuration_diff	IS	-23.99	28.39	-0.85	-0.095	0.12	0.40	0.91
sleepEfficiency_diff	M10	-0.93	1.11	-0.84	-0.11	0.011	0.40	0.91
sleepEfficiency_diff	time1000lastave	-0.21	0.29	-0.73	-0.14	8.65e-03	0.47	0.91
sleepEfficiency_diff	time1lastave	0.17	0.34	0.50	0.069	5.24e-03	0.62	0.91
sleepDuration_diff	time1000lastave	-1.08	2.23	-0.48	-0.086	0.11	0.63	0.91
sleepEfficiency_diff	IV	1.99	4.91	0.41	0.049	4.21e-03	0.69	0.91
sleepDuration_diff	L5	4.40	12.84	0.34	0.042	0.11	0.73	0.91
sleepDuration_diff	time1lastave	-0.73	2.59	-0.28	-0.037	0.11	0.78	0.91
sleepDuration_diff	IV	-4.86	37.28	-0.13	-0.015	0.11	0.90	0.97
sleepEfficiency_diff	L5	-0.023	1.69	-0.014	-1.80e-03	2.22e-03	0.99	0.99

Variables: sleepDuration_diff, absolute of (Sleep diary sleep duration – Fitbit sleep duration); sleepEfficiency_diff, absolute of (Sleep diary sleep efficiency – Fitbit sleep efficiency); TAT250, Duration above 250 lux melanopic EDI (min); M10, Mean light exposure during the 10 brightest consecutive hours (log lux melanopic EDI); L5, Mean light exposure during the 5 dimmest consecutive hours (log lux melanopic EDI); IS, Interdaily stability; IV, Intradaily variability; time1000lastave, Average time of last exposure >1000 lux (decimal hour); time1lastave, Average time of last exposure >1 lux (decimal hour).

Supplementary Table 10. Sociodemographic and health variables of the participants (n = 89).

	First Cohort<br>n = 55	Second Cohort<br>n = 34	Overall<br>n = 89
**Age, n (%)**			
18 - 25	13 (23.64%)	21 (61.76%)	34 (38.20%)
26 - 30	21 (38.18%)	5 (14.71%)	26 (29.21%)
31 - 35	13 (23.64%)	5 (14.71%)	18 (20.22%)
36 - 40	4 (7.27%)	2 (5.88%)	6 (6.74%)
41 - 45	1 (1.82%)	–	1 (1.12%)
46 - 50	1 (1.82%)	1 (2.94%)	2 (2.25%)
51 - 55	1 (1.82%)	–	1 (1.12%)
Over 65	1 (1.82%)	–	1 (1.12%)
**Sex, n (%)**			
Female	27 (49.09%)	18 (52.94%)	45 (50.56%)
Male	28 (50.91%)	16 (47.06%)	44 (49.44%)
**Work, n (%)**			
Full-time	30 (54.54%)	21 (61.76%)	51 (57.30%)
Part-time or student	25 (45.46%)	11 (32.35%)	36 (40.45%)
Non-employed	–	2 (5.89%)	2 (2.24%)
**Smoker, n (%)**			
No	44 (80%)	30 (88.24%)	74 (83.15%)
Yes	11 (20%)	4 (11.76%)	15 (16.85%)
**Health, n (%)**			
Very poor	1 (1.82%)	–	1 (1.12%)
Poor	2 (3.64%)	–	2 (2.25%)
Fair	4 (7.27%)	6 (17.65%)	10 (11.24%)
Good	36 (65.45%)	17 (50%)	53 (59.55%)
Very good	12 (21.82%)	11 (32.35%)	23 (25.84%)
**Disorder, n (%)**			
Color blindness	2 (3.64%)	2 (5.88%)	4 (4.49%)
ADHD	3 (5.46%)	1 (2.94%)	4 (4.49%)
Anxiety/Depression	10 (16.36%)	4 (11.76%)	14 (15.72%)
Migraine	1 (1.82%)	4 (11.76%)	5 (5.61%)
**MSFsc, mean ± SD**	5.02 ± 1.40	4.24 ± 1.00	4.72 ± 1.31
**PROMIS Sleep Disturbance Score, mean ± SD**	–	18.88 ± 6.24	18.88 ± 6.24
**PROMIS Sleep Impairment Score, mean ± SD**	–	16.66 ± 4.16	16.66 ± 4.16
**PSQI score, mean ± SD**	6.29 ± 1.88	–	6.29 ± 1.88

Abbreviations; MSFsc: Mid-Sleep on Free days corrected for sleep debt, PROMIS: Patient-Reported Outcome Measurement Information System, SD: Standard Deviation, PSQI: Pittsburgh Sleep Quality Index
